# Supplementary material for: Deconvolution of cargo delivery and immunogenicity following intranasal delivery of mRNA lipid nanoparticle vaccines
Source: Mol Ther Nucleic Acids. 2025 Apr 24;36(2):102547. doi: 10.1016/j.omtn.2025.102547 (PMC12138548; doi:10.1016/j.omtn.2025.102547)
Supplement: Document S1. Figures S1–S9 and Table S1 [file mmc1.pdf]

OMTN, Volume *36*

Supplemental information

Deconvolution of cargo delivery and immunogenicity following intranasal delivery of mRNA lipid nanoparticle vaccines

Mai N. Vu, Devaki Pilapitiya, Andrew Kelly, Marios Koutsakos, Stephen J. Kent, Jennifer A. Juno, Hyon-Xhi Tan, and Adam K. Wheatley

SUPPLEMENTAL MATERIALS

a

Cationic Lipid	Ionizable Lipid	Helper Lipid	Cholesterol	PEG-Lipid	Lipid ratio*	N:P ratio
- DOTAP	ALC-0315	DSPC	Cholesterol	ALC-0159	46.3/9.4/42.7/1.6	1:5
	SM-102	DSPC	Cholesterol	DMG-PEG2000	50.0/10.0/38.5/1.5	1:5
	Dlin-MC3-DMA	DSPC	Cholesterol	DSPE-PEG2000	52.0/8.0/38.5/1.5	1:5
+ DOTAP (50%)	SM-102	DSPC	Cholesterol	DMG-PEG2000	25.0/5.0/19.2/0.8	1:6
	Dlin-MC3-DMA	DSPC	Cholesterol	DSPE-PEG2000	25.0/5.0/19.2/0.8	1:6

*Ionizable lipid/ Helper lipid/ Cholesterol/ PEG-lipid mole ratio

b

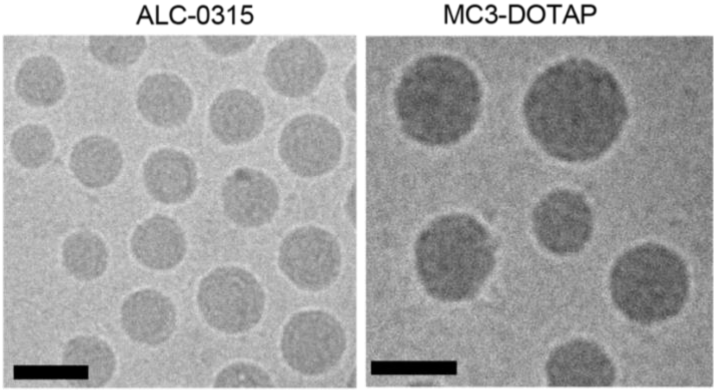

Figure S1: **a**). Formulations of mRNA LNPs. **b**). Representative cryoEM images of ALC-0315 and MC3-DOTAP OVA mRNA LNPs, scale bars = 50 nm.

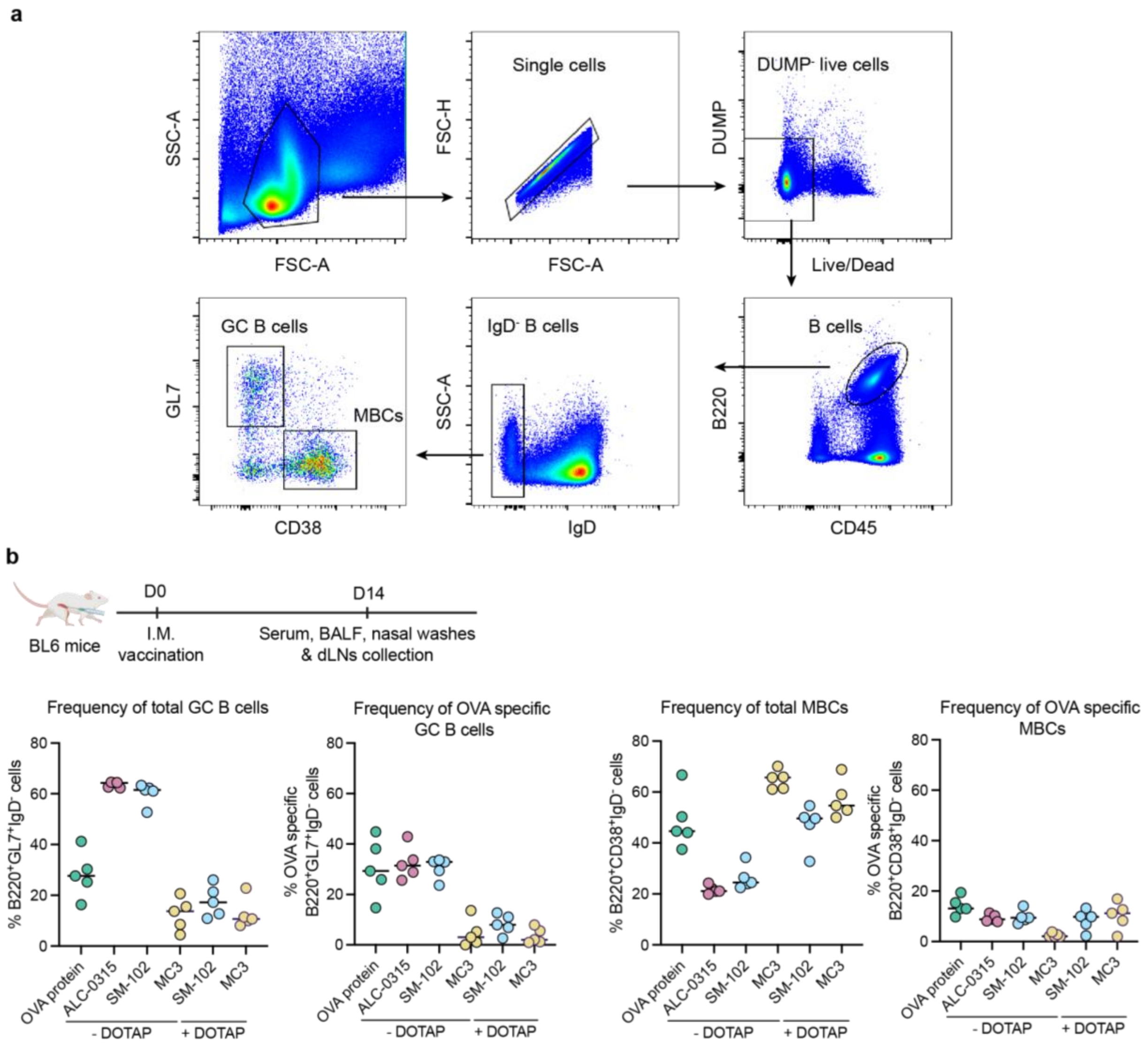

Figure S2: **a**). Gating strategies to identify GC B cells (B220+IgD−GL7+) and memory B cells (B220+IgD−CD38+) in lymph nodes and lungs. **b**). Frequency of total and OVA-specific GC B cells and memory B cells in dLNs at day 14 post i.m. vaccination of OVA protein and OVA mRNA-LNPs (related to figure 1).

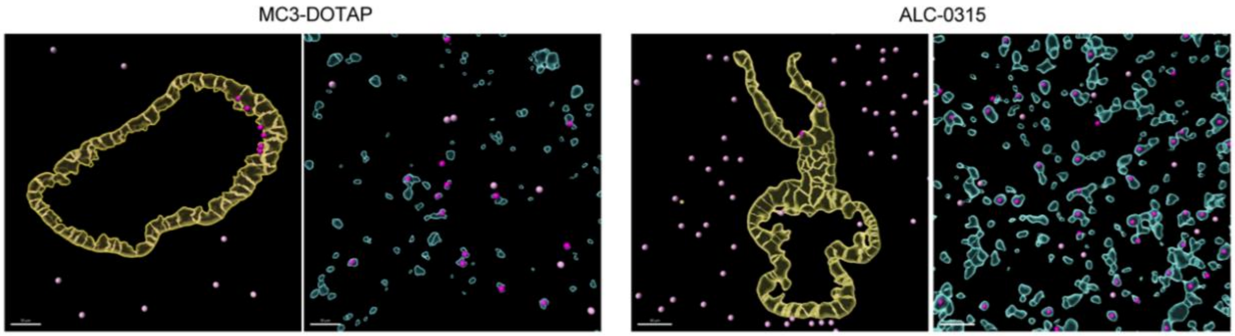

Figure S3: The Spots Close to Surface XTension in Imaris was used to determine the number of tdTom-expressed epithelial (yellow) or immune (cyan) cells. The cells with tdTom spots located inside a 10-thredshold regions (dark magenta) from EpCAM or CD45 staining surfaces were considered tdTom positive cells. In contrast, the cells with tdTom spots located outside the 10-thredshold regions (light magenta) were considered tdTom negative cells.

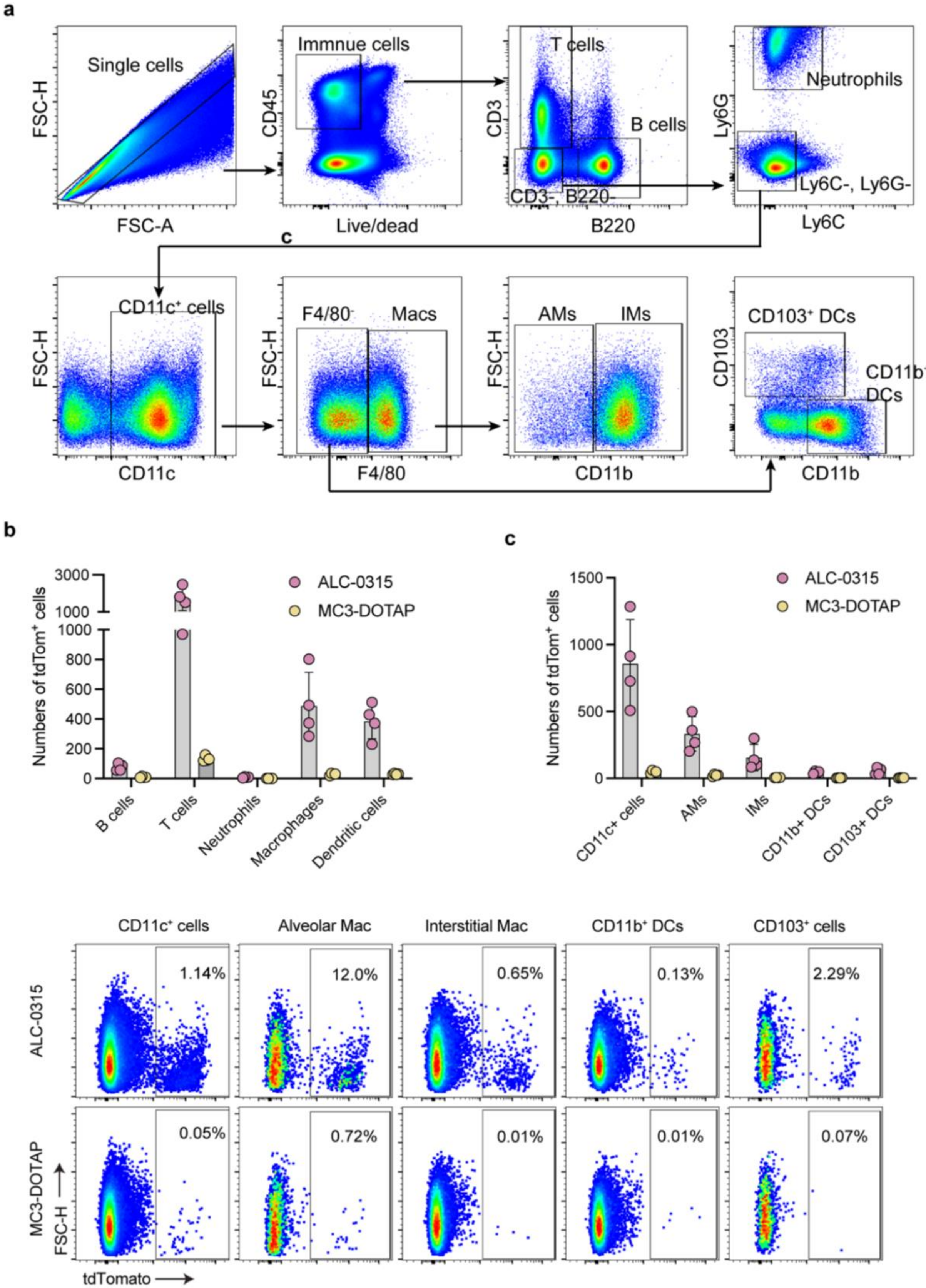
a
Single cells
FSC-H
FSC-A
Immnue cells
CD45
Live/dead
T cells
B cells
CD3-, B220-
CD3
B220
Neutrophils
Ly6C-, Ly6G-
Ly6G
Ly6C
c
CD11c+ cells
FSC-H
CD11c
F4/80-
Macs
F4/80
AMs
IMs
CD11b
CD103+ DCs
CD11b+ DCs
CD103
CD11b
b
Numbers of tdTom+ cells
3000
1000
1000
800
600
400
200
0
ALC-0315
MC3-DOTAP
B cells
T cells
Neutrophils
Macrophages
Dendritic cells
c
Numbers of tdTom+ cells
1500
1000
500
0
ALC-0315
MC3-DOTAP
CD11c+ cells
AMs
IMs
CD11b+ DCs
CD103+ DCs
CD11c+ cells
Alveolar Mac
Interstitial Mac
CD11b+ DCs
CD103+ cells
ALC-0315
1.14%
12.0%
0.65%
0.13%
2.29%
MC3-DOTAP
0.05%
0.72%
0.01%
0.01%
0.07%
FSC-H
tdTomato

Figure S4: **a**). Gating strategies to identify subtypes of immune cells in lungs following CRE mRNA-LNP intranasal vaccination, including CD45+ immune cells, CD3+ T cells, B220+ B cells, Ly6G+ neutrophils, CD11c+ F4/80+ macrophages with CD11b+ interstitial macrophages (IMs) and CD11b- alveolar macrophages (AMs), CD11c+ F4/80- dendritic cells (DCs) with CD11b+ DCs and DC103+ DCs. **b**). Numbers of tdTom positive cells in different CD45+ immune cell subsets in lungs. **c**). Numbers of tdTom positive cells in different dendritic and macrophage cell subtypes (top) and representative images showing tdTom positive cell populations in different dendritic and macrophage cell subsets in lungs (bottom).

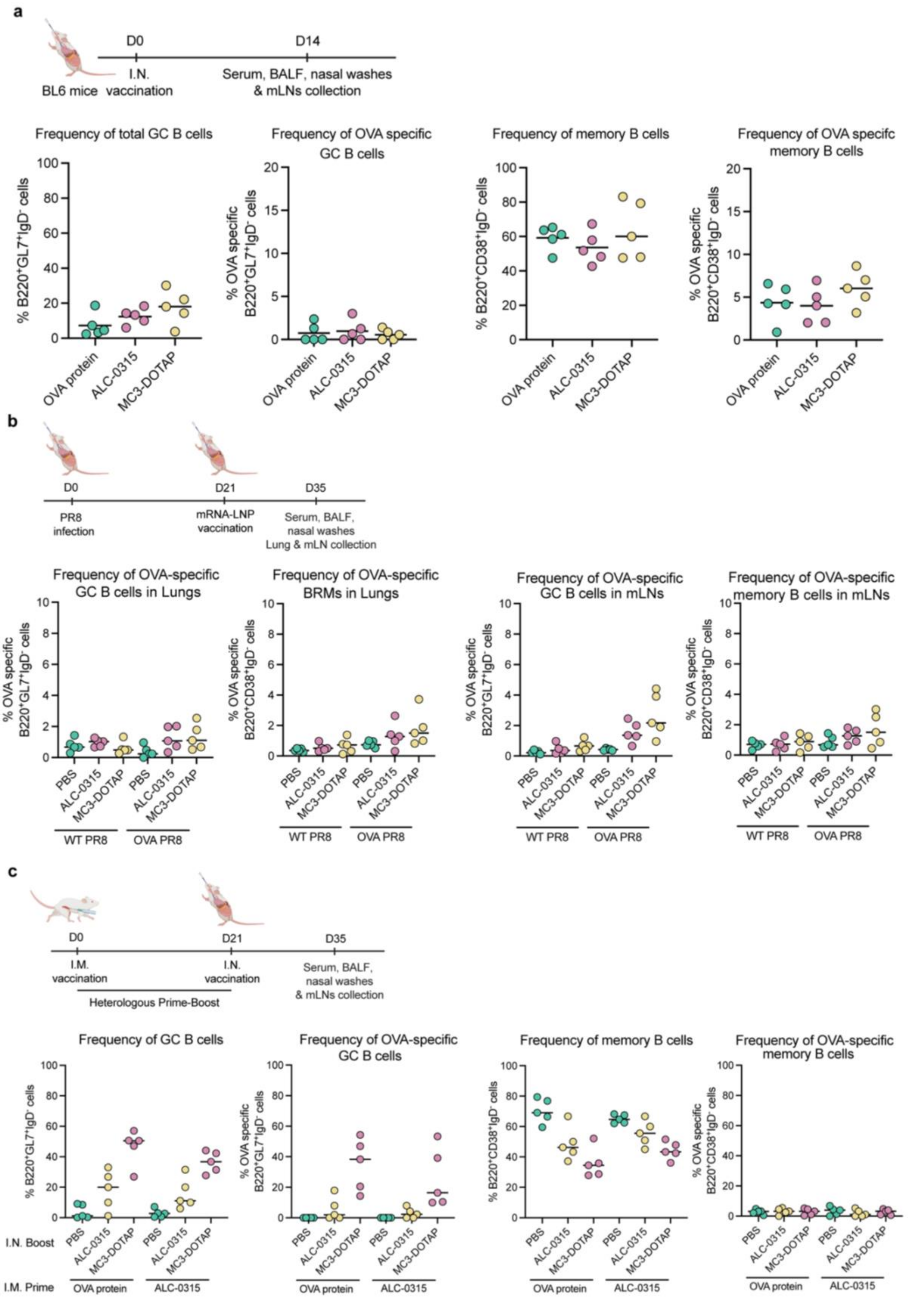
a
D0
D14
BL6 mice
I.N. vaccination
Serum, BALF, nasal washes & mLNs collection
Frequency of total GC B cells
Frequency of OVA specific GC B cells
Frequency of memory B cells
Frequency of OVA specifc memory B cells
% B220+GL7+IgD- cells
% OVA specific B220+GL7+IgD- cells
% B220+CD38+IgD- cells
% OVA specific B220+CD38+IgD- cells
OVA protein
ALC-0315
MC3-DOTAP
b
D0
D21
D35
PR8 infection
mRNA-LNP vaccination
Serum, BALF, nasal washes Lung & mLN collection
Frequency of OVA-specific GC B cells in Lungs
Frequency of OVA-specific BRMs in Lungs
Frequency of OVA-specific GC B cells in mLNs
Frequency of OVA-specific memory B cells in mLNs
PBS
WT PR8
OVA PR8
c
D0
D21
D35
I.M. vaccination
I.N. vaccination
Heterologous Prime-Boost
Serum, BALF, nasal washes & mLNs collection
Frequency of GC B cells
Frequency of OVA-specific GC B cells
Frequency of memory B cells
Frequency of OVA-specific memory B cells
I.N. Boost
I.M. Prime

Figure S5: Frequencies of GC B cells and memory B cells after mRNA-LNP vaccination. **a**). Groups of C57BL/6 (n = 5) were i.n. vaccinated with 2.0 μg OVA protein or OVA mRNA LNPs at 2.0 μg mRNA (top) and frequencies of total and OVA-specific GC B cells and MBCs in mLNs at day 14 post vaccination (bottom) (related to figure 4). **b**). Groups of C57BL/6 (n = 5) were i.n. infected with either WT PR8 at 100 pfu or OVA PR8 at $10^{5.5}$ pfu. At day 21 post infection, mice were i.n. vaccinated with MC3-DOTAP, ALC-0315 OVA mRNA LNPs at 2.0 μg mRNA or PBS control (top) and frequencies of OVA-specific GC B cells and memory B cells in lungs and mLNs at day 14 post boost (bottom) (related to figure 5). **c**). Groups of C57BL/6 (n = 5) were i.m. primed with either 5 μg OVA protein plus 50% volume of Addavax, or ALC-0315 OVA mRNA LNPs at 5 μg mRNA and then i.n. boosted with either ALC-0315, MC3-DOTAP OVA mRNA LNPs at 2.0 μg or PBS control (top) and frequencies of total and OVA-specific GC B cells and MBCs in mLNs at day 14 post boost (bottom) (related to Fig. 6).

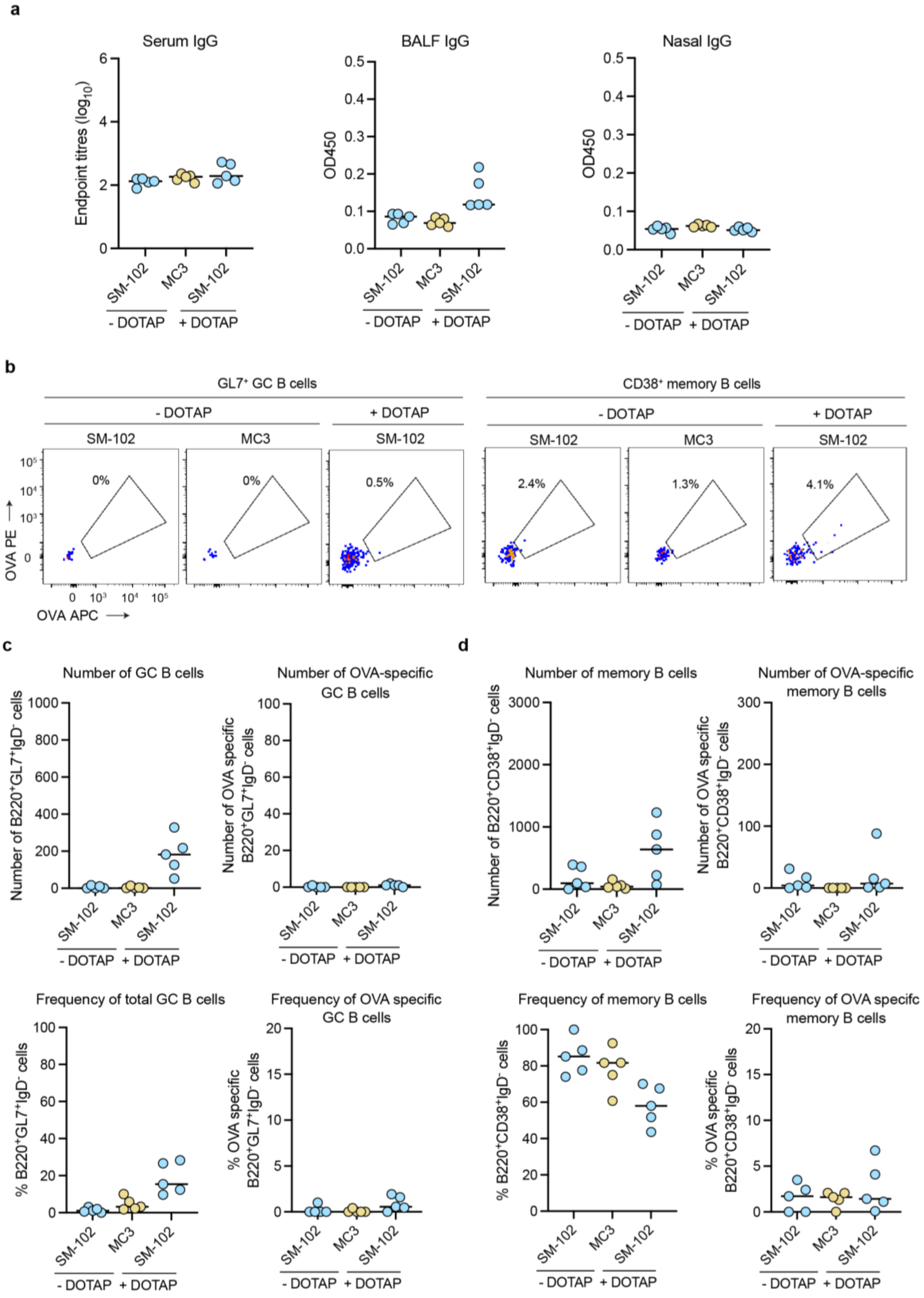
a
Serum IgG
Endpoint titres (log10)
BALF IgG
OD450
Nasal IgG
OD450
SM-102
MC3
SM-102
- DOTAP
+ DOTAP
b
GL7+ GC B cells
CD38+ memory B cells
- DOTAP
+ DOTAP
SM-102
MC3
SM-102
0%
0%
0.5%
2.4%
1.3%
4.1%
OVA PE
OVA APC
c
Number of GC B cells
Number of B220+GL7+IgD- cells
Number of OVA-specific GC B cells
Number of OVA specific B220+GL7+IgD- cells
Frequency of total GC B cells
% B220+GL7+IgD- cells
Frequency of OVA specific GC B cells
% OVA specific B220+GL7+IgD- cells
d
Number of memory B cells
Number of B220+CD38+IgD- cells
Number of OVA-specific memory B cells
Number of OVA specific B220+CD38+IgD- cells
Frequency of memory B cells
% B220+CD38+IgD- cells
Frequency of OVA specifc memory B cells
% OVA specific B220+CD38+IgD- cells

Figure S6: Systemic and mucosal antibody responses to SM-102, MC3, and SM-102-DOTAP OVA mRNA LNPs at 14 days post i.n. vaccination. Groups of C57BL/6 (n = 5) were i.n. vaccinated with the mRNA LNPs at 2.0 μg mRNA. **a**). OVA-specific IgG titers measured in mouse sera, BALF, and nasal washes using ELISA assays. **b**). Representative flow plot of OVA-specific GC and memory B cell populations in mLNs. **c**). Numbers and frequencies of total and OVA-specific GC B cells. **d**). Numbers and frequencies of total and OVA-specific memory B cells.

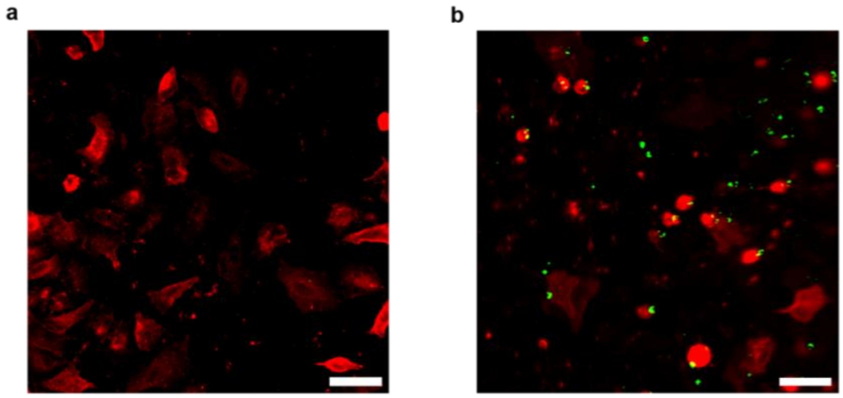

Figure S7: Expression of influenza hemagglutinin (HA; red) or ovalbumin (OVA; green) was assessed in A549 cells at 48h post-infection using immunofluorescent microscopy. **a**). Cells infected with A/Puerto Rico/08/1934 virus alone (MOI of 5). **b**). Cells infected with PR8 expressing OVA (MOI of 1). Scale bars = 100 μm.

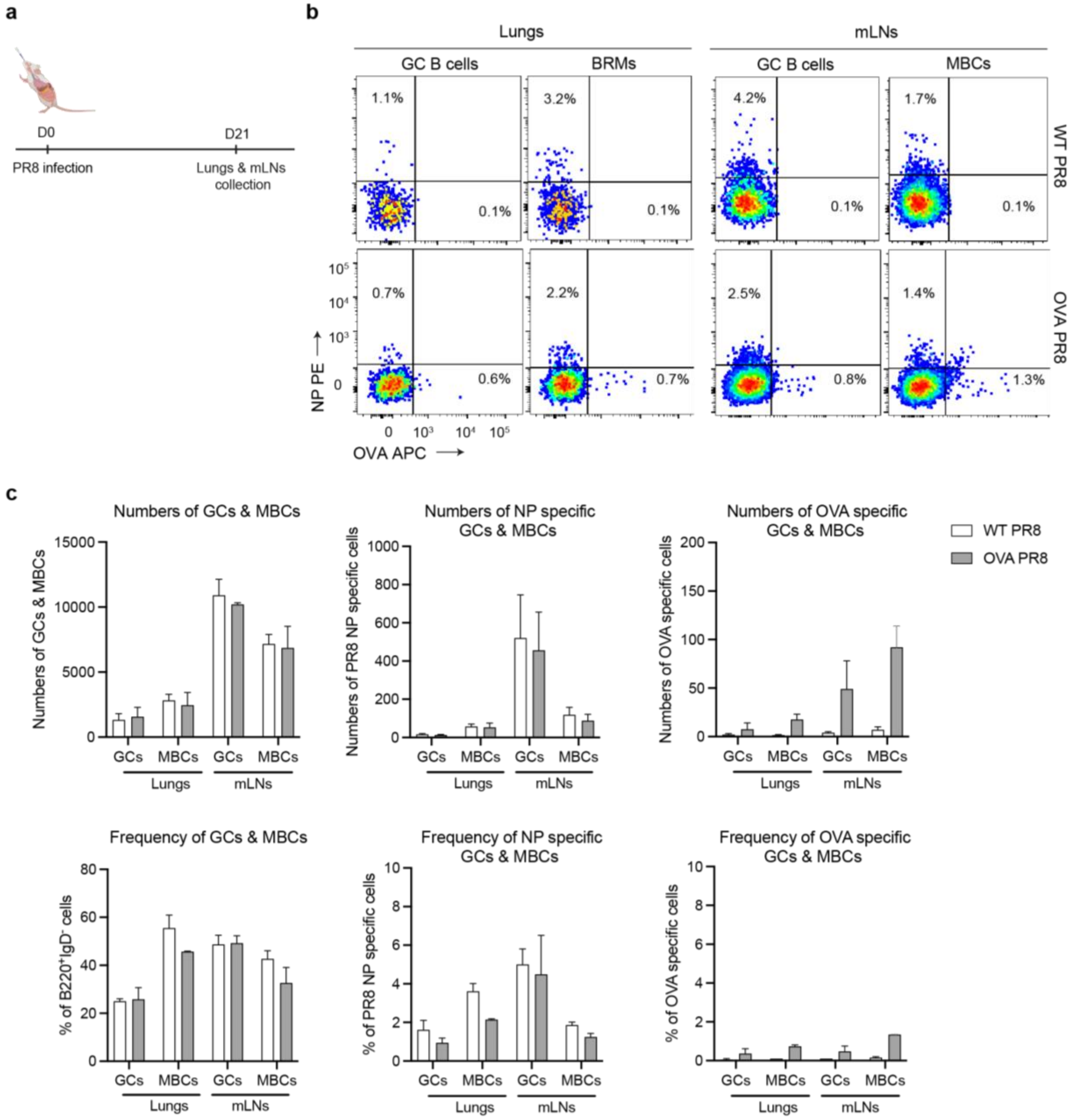

Figure S8: Establishment of antigen-specific B cell population in lungs and mLNs following OVA PR8 infection. **a**). Groups of C57BL/6 were i.n. infected with WT OVA PR8 at $10^{5.5}$ pfu. At day 21 post infection, lungs and mLNs were harvested to assess B cell responses. **b**). Representative flow plot flu- (NP-) or OVA-specific GC B cells and memory B cells in lungs and mLNs. **c**). Numbers and frequencies of total, NP- and OVA-specific GC B cells and memory B cells in lungs and mLNs.

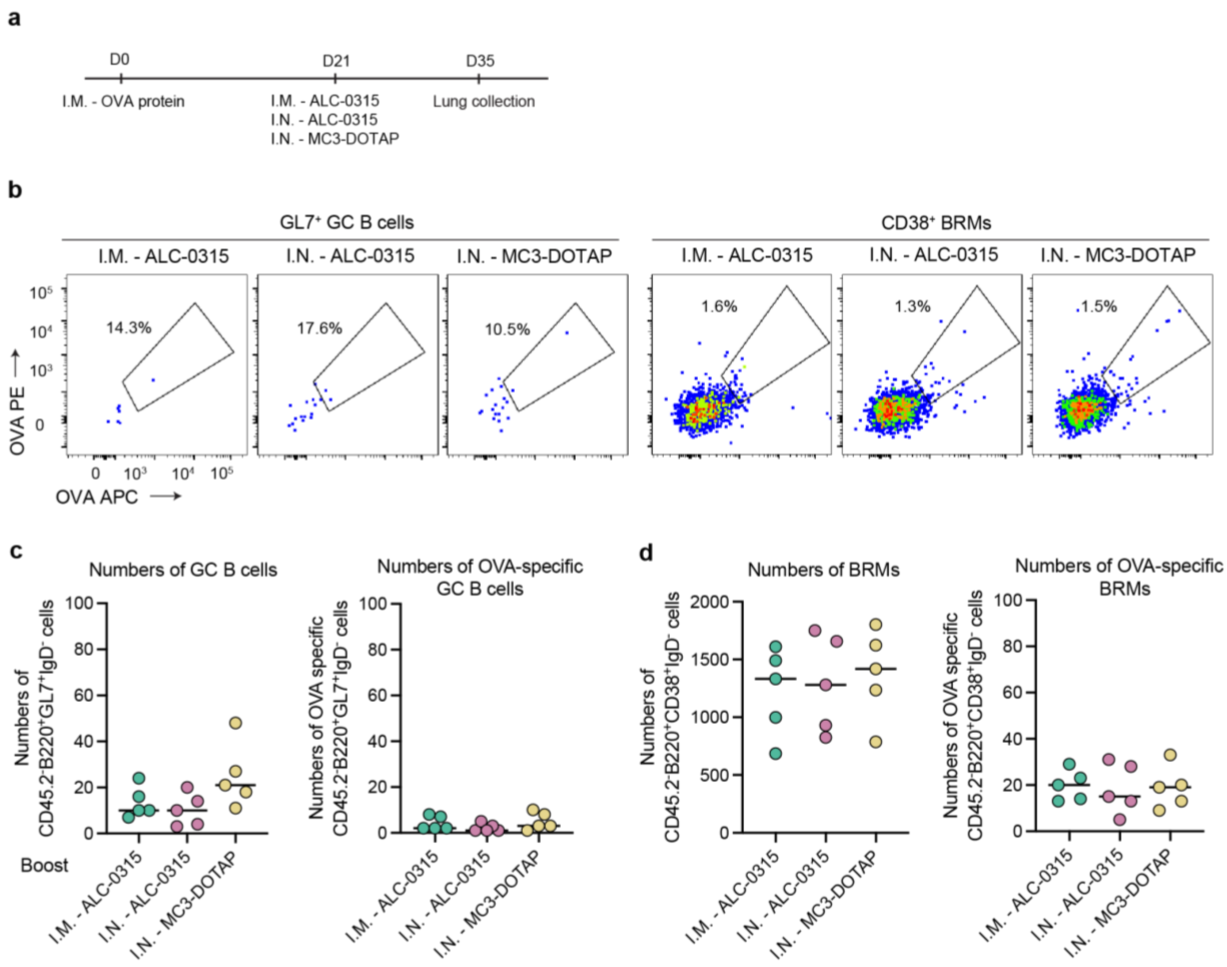

Figure S9: Minimal establishment of tissue-resident B cell responses in lungs following mRNA LNP intranasal vaccination in animals with pre-existing systemic immunity. **a**). Groups of C57BL/6 (n = 5) were i.m. primed with 5 μg OVA protein plus 50% volume of Addavax and 21 days later, i.n. boosted with OVA mRNA LNPs at 2.0 μg mRNA or i.m. boosted with ALC-0315 at 5 μg mRNA as a control. **b**) Representative flow blots of OVA-specific GC B cells and BRMs in lungs at 14 days post boost. **c**). Numbers of total and OVA-specific CD45.2-B220+IgD−GL7+ GC B cells (left) and numbers of total and OVA-specific CD45.2-B220+IgD−CD38+ BRMs (right).

Table S1: Delivery efficiency and immunogenicity of mRNA-LNPs via intramuscular and intranasal administration

**Route**		**I.M.**		**I.N.**	
Formulation		Delivery	Immunogenicity	Delivery	Immunogenicity
- DOTAP	ALC-0315	+++	+++	+++	+
	SM-102	+++	+++	+++	+
	MC3	+	+	+	+
+ DOTAP	SM-102	++	++	++	++
	MC3	+	+	++	++
