# Supplementary material for: Deconvolution of cargo delivery and immunogenicity following intranasal delivery of mRNA lipid nanoparticle vaccines
Source: Mol Ther Nucleic Acids. 2025 Apr 24;36(2):102547. doi: 10.1016/j.omtn.2025.102547 (PMC12138548; doi:10.1016/j.omtn.2025.102547)
Supplement: Document S2. Article plus supplemental information [file mmc2.pdf]

Molecular Therapy
Nucleic Acids
Original Article

Deconvolution of cargo delivery and immunogenicity following intranasal delivery of mRNA lipid nanoparticle vaccines

Mai N. Vu,[1] Devaki Pilapitiya,[1] Andrew Kelly,[1] Marios Koutsakos,[1] Stephen J. Kent,[1,2] Jennifer A. Juno,[1] Hyon-Xhi Tan,[1] and Adam K. Wheatley[1]

[1]Department of Microbiology and Immunology, University of Melbourne, at The Peter Doherty Institute for Infection and Immunity, Melbourne, VIC 3000, Australia; [2]Melbourne Sexual Health Centre and Department of Infectious Diseases, Alfred Hospital and Central Clinical School, Monash University, Melbourne, VIC, Australia

Intranasal vaccination aims to elicit mucosal immunity in the respiratory tract to better protect against respiratory infections (e.g., SARS-CoV-2 and influenza). Most vaccines, including recent COVID-19 mRNA lipid nanoparticles (LNPs), are optimized for intramuscular (i.m.) administration and typically perform poorly when delivered intranasally. Here, we prepared mRNA-LNPs using clinically approved ionizable lipids (ALC-0315, SM-102, and DLin-MC3-DMA) with or without a permanent cationic lipid (1,2-dioleoyl-3-trimethylammonium-propane [DOTAP]) to deliver a model immunogen (ovalbumin [OVA]) and CRE recombinase reporter mRNA. Using wild-type C57BL/6 and Ai14 reporter mouse models, we deconvoluted the effects of LNP formulation on mRNA cargo delivery and immunogenicity following i.m. or intranasal (i.n.) administration. After i.m. vaccination, mRNA-LNPs demonstrated transfection of muscle and immune cells *in vivo*, and consequently robust humoral immune responses. In contrast, mRNA-LNP delivery to the respiratory mucosa was poorly immunogenic, both in naive animals and in those with post-infection inflammation. Encouragingly, mRNA-LNPs efficiently transfected epithelial and immune cells within the lungs and expressed mRNA cargo could efficiently recall immunity in draining secondary lymphoid tissues. The addition of DOTAP led to enhanced recall responses. Decoding interplays of LNP formulations and their performance *in vivo* within specific tissue compartments will provide principles that can guide the rational design of mRNA-LNPs for maximal protection against respiratory diseases.

INTRODUCTION

The ongoing COVID-19 pandemic has underscored the critical need for effective vaccines that not only provide protection against disease but also prevent person-to-person transmission. While intramuscular (i.m.) vaccination with mRNA-based COVID-19 vaccines elicits potent systemic immunity and provides robust protection against severe disease, the induction of mucosal immune responses at sites of viral entry is limited.[1,2] Intranasal (i.n.) vaccination, which potentially induces mucosal antibodies (IgA and IgG) and tissue-resident memory T and B (BRM) cells, might help to prevent infection, control viral replication, and reduce transmission.[3–7] However, attempts to develop i.n. COVID-19 vaccines using delivery platforms such as viral vectors, live-attenuated viruses, and recombinant proteins have had limited clinical success to date.[8–10] mRNA-lipid nanoparticles (LNPs) are a potential novel platform for i.n. vaccine delivery; however, both the utility and safety have yet to be comprehensively established.[11]

Current LNP formulations generally comprise four components: an ionizable lipid, a polyethylene glycol (PEG)-conjugated lipid, cholesterol, and a "helper" lipid. The ionizable lipid plays a critical role in mRNA cargo delivery and subsequent immune responses, as its positive charge at low pH enables efficient encapsulation of negatively charged mRNA and allows endosomal escape for mRNA release into the cytosol.[12] Additionally, ionizable lipids exhibit an adjuvant effect, contributing to robust immunogenicity following i.m. vaccination.[13] However, i.n. administration of currently licenced mRNA-LNP vaccines induce minimal immunity in non-human primate models,[14] suggesting barriers exist to the efficient generation of vaccine-elicited immunity within the respiratory mucosa. Interestingly, the use of positively charged materials (e.g., DOTAP, polyethylenimine [PEI], and chitosan) in vaccine formulations appears to improve mucosal delivery.[5,15] For example, modification of the COVID-19 vaccine mRNA-1273 (Spikevax; Moderna) through the incorporation of a permanent cationic lipid restored antibody responses when administrated intranasally and conferred protection against viral challenge.[16] However, the immunobiological mechanisms underlying the induction of local immunity following i.n. administration of mRNA vaccines with or without the presence of the cationic lipid remained unclear.

Here, we aimed to deconvolute the processes of mRNA cargo delivery and biogenesis of the immune response within the respiratory

Received 28 December 2024; accepted 22 April 2025;
https://doi.org/10.1016/j.omtn.2025.102547.

Correspondence: Adam K. Wheatley, Department of Microbiology and Immunology, University of Melbourne, at The Peter Doherty Institute for Infection and Immunity, Melbourne, VIC 3000, Australia.
E-mail: a.wheatley@unimelb.edu.au

© 2025 The Author(s).
Published by Elsevier Inc. on behalf of The American Society of Gene and Cell Therapy.
This is an open access article under the CC BY license (http://creativecommons.org/licenses/by/4.0/).

Table 1. Characterization of OVA mRNA-LNPs, including nanoparticle diameter, polydispersity index, zeta potential, and encapsulation efficiency

Cationic lipid	Ionizable lipid	Diameter (nm)	PdI	Zeta potential (mV)	Encapsulation efficiency (%)
−DOTAP	ALC-0315	60.5 ± 1.3	0.092	−7.97	87.8
	SM-102	76.4 ± 1.7	0.097	−8.38	88.4
	MC3	74.9 ± 2.4	0.166	−6.1	88.8
+DOTAP	SM-102	120.3 ± 5.1	0.129	+12.3	98.6
	MC3	125.3 ± 3.4	0.134	+14.5	94.2

mucosa and associated lymphoid sites. We contrasted the immunogenicity of mRNA-LNPs formulated using clinically approved ionizable lipids (ALC-0315, SM-102, and MC3) either alone or with the addition of a cationic lipid DOTAP. When mRNA-LNPs were administered intramuscularly, the efficiency of *in vivo* mRNA cargo delivery was associated with robust vaccine immunogenicity. However, in the lung following i.n. administration, mRNA delivery efficiency and immunogenicity were decoupled. Despite high transfection efficiency in lung cells, immune responses to i.n. mRNA-LNP vaccination were limited. In line with a previous study that demonstrated the outperformance of using self-amplifying mRNA as an i.n. booster, particularly in improved mucosal immunity,[17] here we found that i.n. boosting with mRNA-LNP vaccines could efficiently recall both systemic and mucosal immunity. Our study highlights that unlocking the promise of mucosal immunization strategies to combat respiratory pathogens will require a greater understanding of the biogenesis of mucosal immunity and advances in LNP formulations.

RESULTS

LNP formulation influences humoral immune responses following i.m. vaccination

Prototypic four lipid-component formulations for i.m. delivery of mRNA-LNP vaccines have been comprehensively established for mRNA COVID-19 vaccines. As the addition of the fifth cationic lipid DOTAP in a modified Onpattro formulation (MC3-DOTAP) has been reported to selectively target LNPs to the lungs following intravenous (i.v.) injection,[18] we evaluated systemic and mucosal immune responses to mRNA vaccines formulated with or without DOTAP.

Here, mRNA-LNPs encoding OVA were prepared by microfluidic mixing methods using the NanoAssemblr Ignite system as described previously.[19] LNPs were formulated analogously to the COVID-19 Comirnaty vaccine (ALC-0315; Pfizer/BioNTech), Spikevax vaccine (SM-102; Moderna), Onpattro (MC3; Alnylam Pharmaceuticals), or combining SM-102 or MC3 with DOTAP (Figure S1A). The incorporation of DOTAP led to an increase in LNP size from approximately 60–75 nm (ALC-0315, SM-102, and MC3) to ∼120–125 nm (SM102-DOTAP and MC3-DOTAP) and changed the surface charge of the nanoparticles from negative to positive (Table 1). The morphology of mRNA-LNPs in the absence (ALC-0315) or presence (MC3-DOTAP) of DOTAP was confirmed by representative cryoelectron microscopy (cryo-EM) images (Figure S1B).

We next compared the elicitation of systemic and mucosal immunity following i.m. vaccination with the five OVA mRNA-LNPs versus a conventional OVA protein in adjuvant (Addavax) control. OVA-specific antibody titers were measured in serum, bronchoalveolar lavage fluid (BALF), or nasal wash samples at 14 days post-immunization (dpi) (Figure 1A). A single OVA protein vaccination elicited moderate titers of specific serum IgG (endpoint dilutions $\sim 10^4$) and detectable IgG in BALF while minimal antibody responses were observed in nasal washes (Figure 1B). Prototypic mRNA-LNP vaccines (ALC-0315 and SM-102) elicited not only robust and comparable OVA-specific IgG responses in serum and BALF but also negligible nasal IgG levels. The incorporation of DOTAP into SM-102 formulation resulted in a reduction in serum IgG titers and a loss in mucosal IgG. Similarly, MC3-formulated vaccines were poorly immunogenic, with the addition of DOTAP failing to augment immunogenicity.

The humoral immune response is potentiated within germinal centers (GCs) formed in draining lymph nodes (dLNs) proximal to the vaccination site, where antigen-experienced B cell undergo somatic hypermutation and affinity maturation.[20] Here, we utilized fluorophore-conjugated OVA protein as B cell probes to quantify GC B cell responses in dLNs (inguinal and iliac LNs). Following a single immunization and in line with serological responses, we found markedly higher numbers of total and OVA-specific GC ($GL7^+B220^+IgD^-$) and memory B cells (MBCs; $CD38^+B220^+IgD^-$) after ALC-0315 and SM-102 vaccination compared to all other vaccine groups (Figures 1C–1E and S2). Again, formulations utilizing MC3 with or without DOTAP as well as SM102-DOTAP were non-immunogenic.

To investigate the impact of formulation upon mRNA delivery, we selected MC3-DOTAP and ALC-0315 as prototype formulations and utilized the Ai14 mouse model[21] to study the transfection efficiency of vaccine mRNA *in vivo* post-vaccination. In this Ai14 mouse line, mRNA delivery of CRE recombinase into cells mediates deletion of a loxP-flanked STOP cassette, resulting in the expression of a tdTomato reporter (tdTom) (Figure 2A). To enable direct visualization of the LNPs post-injection, we additionally labeled LNPs with a fluorescent dye (1,1-Dioctadecyl-3,3,3,3-tetramethylindodicarbocyanine [DiD]).

Ai14 mice were injected intramuscularly and injection sites and dLNs (inguinal lymph nodes) were visualized 48 h after

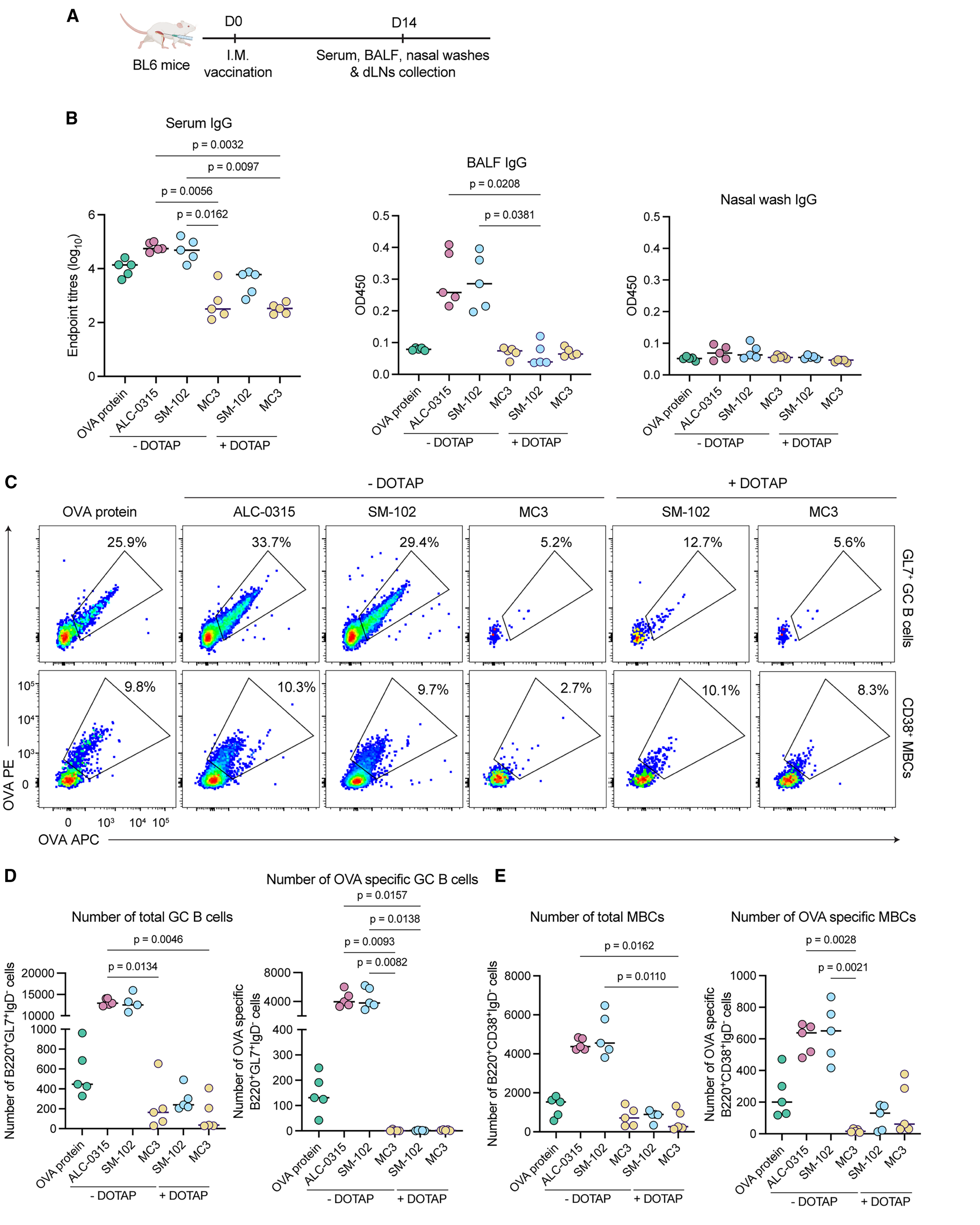

(legend on next page)

immunization by confocal microscopy (Figure 2B). The diffuse localization of LNPs formulated with either MC3-DOTAP or ALC-0315 was observed in both muscle and dLN tissues (Figure 2C). However, only ALC-0315 LNPs efficiently delivered mRNA cargo *in vivo*, with prominent tdTom expression. In the muscle, expression was concentrated in $CD45^+$ immune cells and $laminin^+$ muscle fibers, while in the lymph node, tdTom expression was observed throughout the follicle, subcapsular sinus, and medulla including within $CD11c^+$ dendritic cells (Figure 2C). Poor transfection efficacy in both the muscle and dLNs appeared to underpin the limited immunogenicity of MC3-DOTAP formulated with MC3-DOTAP.

Efficient delivery but limited immunogenicity following i.n. immunization with mRNA-LNP

LNPs formulated with MC3 and 50% DOTAP were previously demonstrated to preferentially deliver mRNA to the lungs after i.v. administration, indicating a degree of mucosal tissue tropism for this formulation, although delivered through the vasculature.[18] We therefore evaluated if i.n. delivery of analogous MC3-DOTAP mRNA-LNPs to the respiratory mucosa resulted in the effective delivery of mRNA cargo compared to the canonical ALC-0315 mRNA-LNPs using the Ai14 mouse model (Figure 3A). Interestingly, both LNPs formulated with MC3-DOTAP and ALC-0315 could efficiently deliver CRE mRNA to lung cells (median 0.65% and 2.95% of total transfected lung cells, respectively [p = 0.0079]) (Figures 3B, 3C, and S3). A closer examination of the tdTom-expressing cell distribution revealed that epithelial cells were found to be selectively transfected by MC3-DOTAP LNPs, accounting for ∼15.8% of total $tdTom^+$ cells. In contrast, few epithelial cells expressed tdTom following ALC-0315 LNP vaccination, accounting for only ∼0.4% of total $tdTom^+$ cells (Figure 3C) with the majority of $tdTom^+$ cells after both ALC-0315 and MC3-DOTAP vaccination being $CD45^+$ immune cells (median 81.3% and 72.6%, respectively). We next further determined $tdTom^+$ expression among immune cell subsets including B cells, T cells, neutrophils, macrophages, and dendritic cells within the lungs using flow cytometry (Figures 3D and S4A). Consistent with the confocal data (Figure 3C), here we found a significantly higher number of $tdTom^+$ $CD45^+$ cells following ALC-0315 compared to MC3-DOTAP administration (p = 0.0286; Figure 3E). Interestingly, both ALC-0135 and MC3-DOTAP LNPs were efficiently taken up by T cells, accounting for approximately 58.4% and 59.5% of total $tdTom^+$ immune cells, respectively. However, compared to MC3-DOTAP, ALC-0315 LNPs induced a markedly higher number of $tdTom^+$ cells across all immune cell subsets examined (Figure S4B) and within subpopulations of macrophages (alveolar macrophages [AMs] and interstitial macrophages [IMs]) and dendritic cells ($CD11b^+$ and $CD103^+$ DCs) (Figure S4C). Overall, we found that both the canonical ALC-0315 and MC3-DOTAP mRNA-LNP were capable of cargo delivery to epithelial and non-epithelial cell populations in the lung, but with ALC-0315 displaying a higher transfection efficiency.

We therefore investigated if MC3-DOTAP and ALC-0315 OVA mRNA-LNPs would induce enhanced mucosal antibody responses compared to controls of OVA protein controls when administrated intranasally (Figure 4A). At day 14 post i.n. immunization, OVA-specific IgG titers were absent in serum for all three groups. Nonetheless, MC3-DOTAP vaccination elicited modest increases in IgG levels in BALF and nasal washes compared to ALC-0315 or OVA protein (Figure 4B). Evaluation of antigen-specific B cell responses in the mediastinal lymph nodes draining the lung did not show an appreciable induction of OVA-specific GC B cells by any of the vaccines (Figures 4C and 4D), with only very low numbers of antigen-specific MBCs generated by the OVA protein and MC3-DOTAP, but not by the ALC-0315 vaccines (Figures 4C–4E and S5A). To generalize the lack of immunogenicity after i.n. delivery of mRNA-LNPs, we confirmed our observations using mRNA-LNPs formulated with SM-102, MC3 alone (without DOTAP), which similarly displayed negligible B cell and antibody responses (Figure S6). Similar to MC3-DOTAP, addition of DOTAP in SM-102 formulation only induced modest BALF IgG and antigen-specific MBCs in mLNs following i.n. vaccination. We also assessed if i.n. delivery of MC3-DOTAP to the respiratory mucosa could elicit local immunity in the lungs; however, minimal B cell immunity was also observed (Figure 4F). Collectively, although both ALC-0135 and MC3-DOTAP displayed the capacity to deliver cargo to the lung via i.n. administration, they failed to elicit robust systemic or local mucosal immune responses.

mRNA-LNP i.n. vaccination fails to recall pre-established B cells in inflamed respiratory tissues

The lung itself is not typically considered an efficient site biogenesis for adaptive immunity, although it is known that inducible bronchus associated lymphoid tissues (iBALT) can be formed after inflammatory stimuli.[22] GC B cells and BRM cells have been reported to be established in the lungs following influenza[23,24] or SARS-CoV-2 infection,[25] forming antigen-specific B cell pools that support rapid antibody production upon re-exposure. We therefore assessed B cell responses following i.n. delivery of OVA mRNA-LNP into inflamed lungs previously exposed to influenza infection with an established iBALT architecture.[23] Mice were first infected with mild-to-moderate sublethal doses of either wild-type A/Puerto Rico/08/1934 influenza (WT PR8) or PR8 viruses with modified non-structural (NS) segments to express OVA protein (OVA PR8).[26] The capacity of the recombinant

Figure 1. Impact of LNP formulation on humoral immune responses after intramuscular vaccination of mRNA vaccines

(A) Groups of C57BL/6 (n = 5) were i.m. vaccinated with 5 μg OVA protein plus 50% volume of Addavax or OVA mRNA LNPs (5 μg mRNA). (B) OVA-specific IgG titers measured in mouse sera, BALF, and nasal washes at day 14 post vaccination using ELISA assays. (C) Representative flow plot of OVA-specific GC B cell populations ($IgD^-B220^+GL7^+$) (top) and memory B cell populations ($IgD^-B220^+CD38^+$) (bottom) in draining LNs at day 14 post vaccination. (D) Numbers of total and OVA-specific GC B cells. (E) Numbers of total and OVA-specific memory B cells. Statistical significance was determined by a Kruskal-Wallis test followed by post-hoc Dunn's multiple comparisons test.

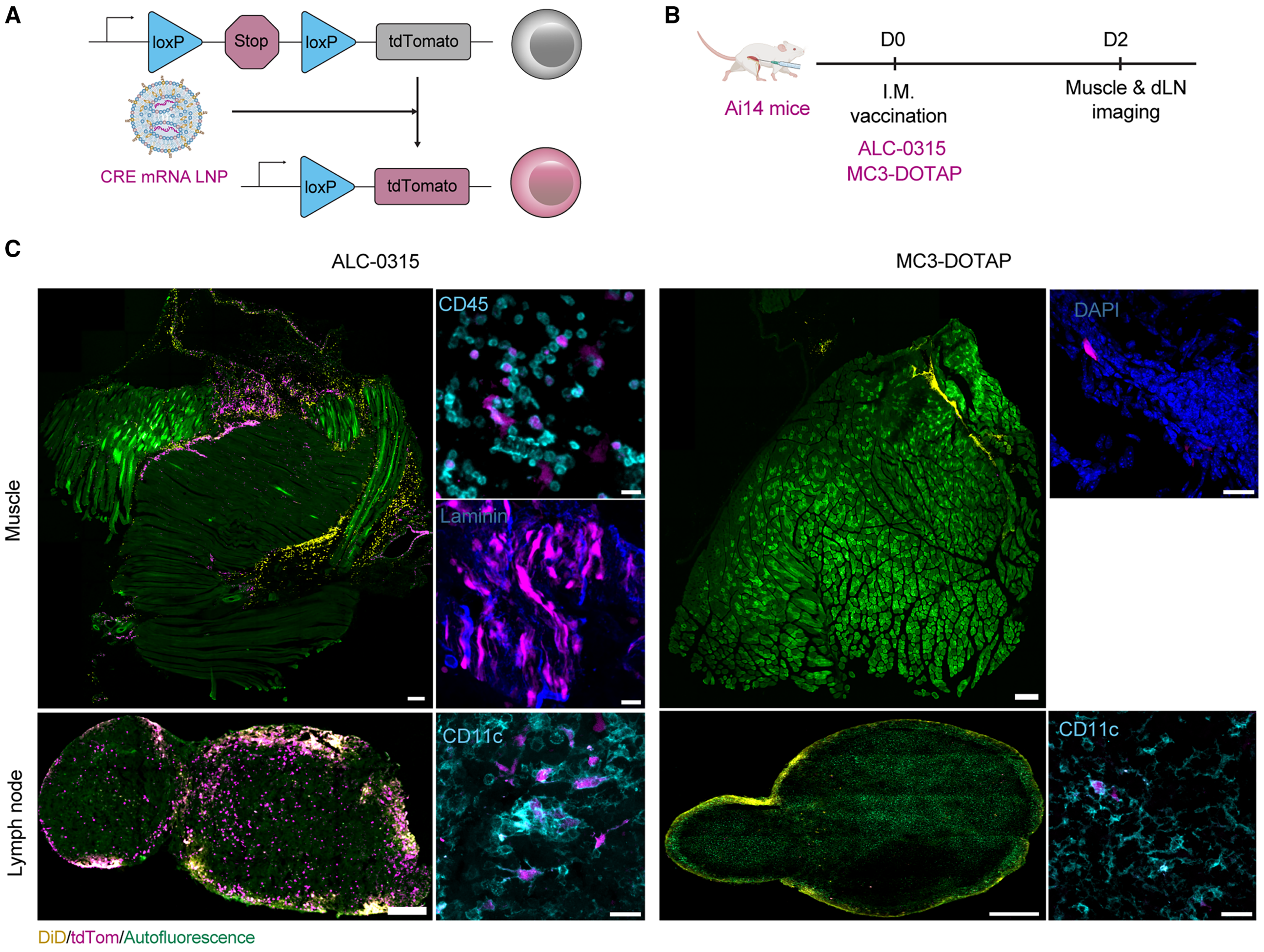

Figure 2. Impact of LNP formulation on cargo delivery after intramuscular vaccination of mRNA vaccines

(A) Diagram of loxP-flanked STOP cassette upstream of tdTomato. Cellular uptake of CRE mRNA-LNPs drives expression of CRE recombinase in Ai14 mice, (B) CRE mRNA LNPs (5.0 μg mRNA) were i.m. administrated into Ai14 mice. (C) Confocal images of muscles at injection sites and draining inguinal lymph nodes at 48 h post injection: tdTomato (magenta), DiD-labeled LNPs (yellow), and autofluorescence (green). For whole tissue images, scale bars: 300 μm; for inset images, scale bars: 20 μm.

influenza viral vector to drive OVA protein expression was confirmed following *in vitro* infection in the A549 human lung epithelial cell line using immunofluorescent microscopy (Figure S7).[27,28] At day 21 after infection, mice were i.n. vaccinated with MC3-DOTAP, ALC-0315 mRNA-LNPs, or PBS control (Figure 5A). Small increases in mucosal IgG levels were observed after vaccination with mRNA-LNP only in mice infected with OVA PR8 (Figure 5B). Despite the evidence of established iBALTs in lungs at day 21 post-infection (Figure S8), we again found limited primary immunogenicity, with no elicitation or significant populations of BRM cells observed (Figures 5C, 5D, and S5B). Overall, these data suggested that pre-existing iBALT immune architecture within the lung was not sufficient to allow biogenesis of mucosal immunity following i.n. delivery of mRNA-LNPs. Encouragingly, in the lung-draining mLNs, we observed a noticeable rise in numbers of antigen-specific GCs and MBCs after mRNA-LNP vaccination exclusively in mice previously infected with OVA PR8 (Figures 5C and 5E), suggesting a potential role of mRNA-LNP i.n. vaccination in recalling pre-existing immunity established in local secondary lymphoid tissues.

Intranasal boosting with MC3-DOTAP can recall systemic and mucosal responses in pre-immune contexts

While i.n. delivery of mRNA-LNP appeared poorly immunogenic as primary immunization, it may still be useful for so-called "prime-pull" strategies[7] that aim to relocate systemically elicited immunity from the circulation and lodge these into the tissues. We therefore assessed whether i.n. boosting of previously immunized animals could further augment mucosal immunity. Mice were primed intramuscularly with OVA protein or ALC-0315 mRNA-LNP, then 21 days later intranasally with MC3-DOTAP, ALC-0315 OVA mRNA-LNP, or PBS control (Figure 6A). Fourteen days after boosting, we observed increased titers of OVA-specific IgG in serum and

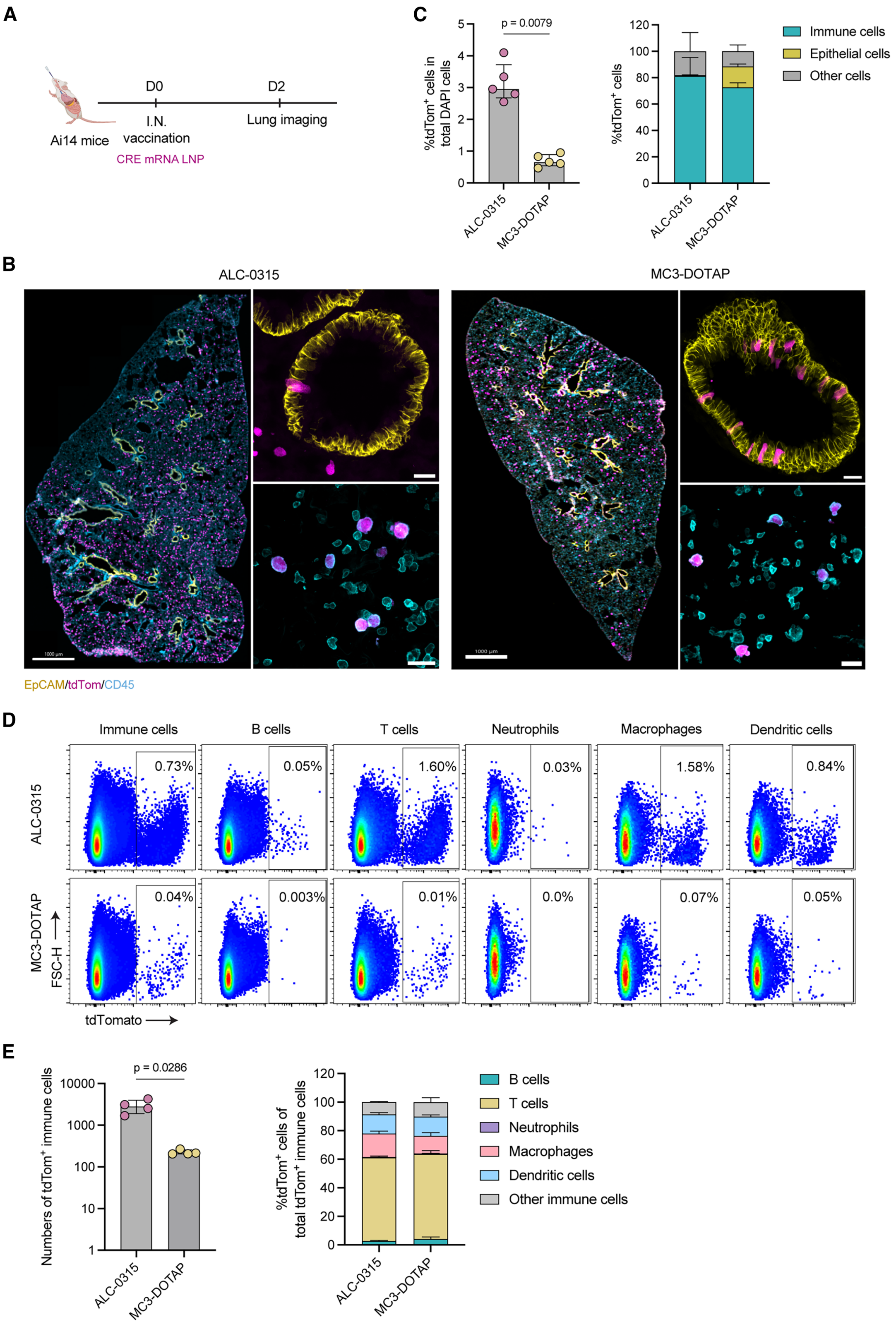

(legend on next page)

BALF as well as minor increases in nasal IgG in mice boosted with MC3-DOTAP compared to ALC-0315 and PBS controls (Figure 6B). Although ALC-0315 induced higher IgG responses after a single i.m. shot compared to OVA protein, there were no significant differences in both systemic and mucosal IgG levels elicited by these two vaccines following MC3-DOTAP i.n. boosting. Boosting with ALC-0315 also slightly improved serum IgG levels and in BALF IgG particularly in OVA protein-vaccinated animals but induced limited antibody in nasal washes.

Within the lung-draining mLNs, we found significant increases in the number and frequencies of OVA-specific GC B cells and MBCs after boosting with MC3-DOTAP (Figures 6C–6E and S5C), with a more modest rise observed for animals receiving ALC-0315 i.n. boosting. While minimal B cell immunity was established in the lungs (Figure S9), collectively these findings suggest that delivery of OVA antigens intranasally using mRNA is sufficient for recalling systemic immunity at secondary lymphoid sites draining the lung.

DISCUSSION

During the COVID-19 pandemic, i.m. administration of mRNA-LNP vaccines demonstrated great effectiveness; however, wider utilization for mucosal vaccine delivery has yet to be established. In animal models, i.n. administration of canonical formulations of mRNA-LNP (Pfizer- and Moderna-like formulations) has met with limited success. Along with the high risk of inflammation associated with the LNP components,[29,30] i.n. vaccination of licensed COVID-19 mRNA vaccines to date has only induced minimal immunogenicity.[14] Here, we similarly found poor elicitation of both mucosal and systemic immunity upon delivery of ALC-0315 or SM-102 mRNA-LNPs via i.n. routes. Despite evidence of robust mRNA transfection in immune cells within the respiratory mucosa, these vaccines were unable to prime an immune response within the lungs or lung-draining lymph nodes in both naive and pre-infected animal models (Table S1). Differential transfection efficiency and targeted cell types were observed in the lung following i.n. administration of ALC-0315 and MC3-DOTAP mRNA-LNPs, with ALC-0315 exhibiting a ~4.5-fold higher number of transfected cells. One factor that might contribute to increased cellular uptake was nanoparticle size, with smaller-sized ALC-0315 LNPs (~60.5 nm) potentially more efficiently taken up into cells compared to larger MC3-DOTAP counterparts (~125.3 nm).[31] While delivery to airway epithelial cells was most efficient with MC3-DOTAP mRNA-LNPs, both formulations were able to deliver cargo to immune cells such as $CD11b^{+}$ and $CD103^{+}$ DCs and drive protein production *in vivo*. Nevertheless, immunogenicity remained minimal.

The incorporation of a cationic lipid DOTAP into a modified Onpattro LNP formulation (MC3-DOTAP) was previously demonstrated to allow efficient delivery of mRNA reporters to the lungs following i.v. injection.[18] Notably this "lung-homing" property was attributed to altering the surface charge of mRNA-LNP, but it was only established for trafficking from circulation to the lungs. In line with prior reports,[32] we found DOTAP containing LNPs ill-suited for conventional vaccine delivery, with limited transfection efficiency in the muscle or dLN and no discernible immunogenicity (Table S1). However, DOTAP mRNA-LNPs were similarly non-immunogenic when delivered intranasally. Again, there was robust transfection of epithelial and immune cells in the lung, but an inability to potentiate immunity, suggesting expression of vaccine antigens alone in the respiratory tract is insufficient to drive the biogenesis of mucosal immune responses. While modification of COVID-19 Spikevax vaccines (Moderna) via incorporation of a permanent cationic lipid did demonstrate a capacity to elicit immune responses when delivered intranasally, immunogenicity remained markedly suboptimal and comparable to that of conventional i.m. delivery that was dosed at ~25- to 60-fold lower.[16]

Despite the limited clinical success,[9,10] i.n. vaccination of viral vector vaccines is usually able to induce high levels of systemic and mucosal antibody (IgG and IgA) as well as tissue-resident B and T cell responses in preclinical animal models.[33–35] However, in the present study, the generation of pre-inflamed lungs with an established iBALT architecture via influenza infection failed to rescue the elicitation of tissue-localized B cell responses to i.n.-delivered mRNA vaccines. Interestingly, we observed increased antigen-specific GC B cells and MBCs in lung-draining mLNs after i.n. mRNA-LNP vaccination in OVA PR8-infected mice, suggesting potential trafficking of antigens or antigen-presenting cells from the lungs to mLNs, that might subsequently recall B cell responses pre-established in the mLNs following influenza infection.

Human populations are recurrently infected and/or vaccinated with a range of respiratory pathogens, including influenza, respiratory syncytial virus, and SARS-CoV-2, with prior immunity to these pathogens now nearly universal. In such environments, mucosal vaccination might serve to expand immunity within the respiratory mucosa to bolster protection against viral acquisition and limit spread. We therefore tested if a "prime & pull" strategy[7] was viable, where pre-existing systemic immunity could be boosted intranasally to seed mucosal immune responses. In this context, i.n. boosting was observed to increase titers of antigen-specific antibodies in both systemic and mucosal compartments, consistent with previous

Figure 3. Impact of LNP formulation on cargo delivery after intranasal vaccination of mRNA vaccines

(A) CRE mRNA LNPs (2.0 μg mRNA) were i.n. administrated into Ai14 mice. (B) Representative images of lungs with tdTom+ cells (magenta), epithelial cells (yellow), and immune cells (cyan) at 48 h after administration. For whole lobe sections, scale bars: 1,000 μm; for inset images, scale bars: 20 μm. (C) Percentage of tdTom positive cells in total DAPI positive cells and contribution of different cell types in total tdTom+ cells. Data of each group were analyzed from 10 individual images taken from 5 mice using Imaris v10. (D) Representative flow plots of tdTom cell populations in different immune cell subsets in lungs. (E) Numbers of total $tdTom^{+}$ $CD45^{+}$ immune cells in lungs (n = 4) (left) and contribution of different cell types in $tdTom^{+}$ $CD45^{+}$ immune cell subsets in lungs (right). Statistical significance was determined by a non-parametric unpaired two-tailed Mann-Whitney test.

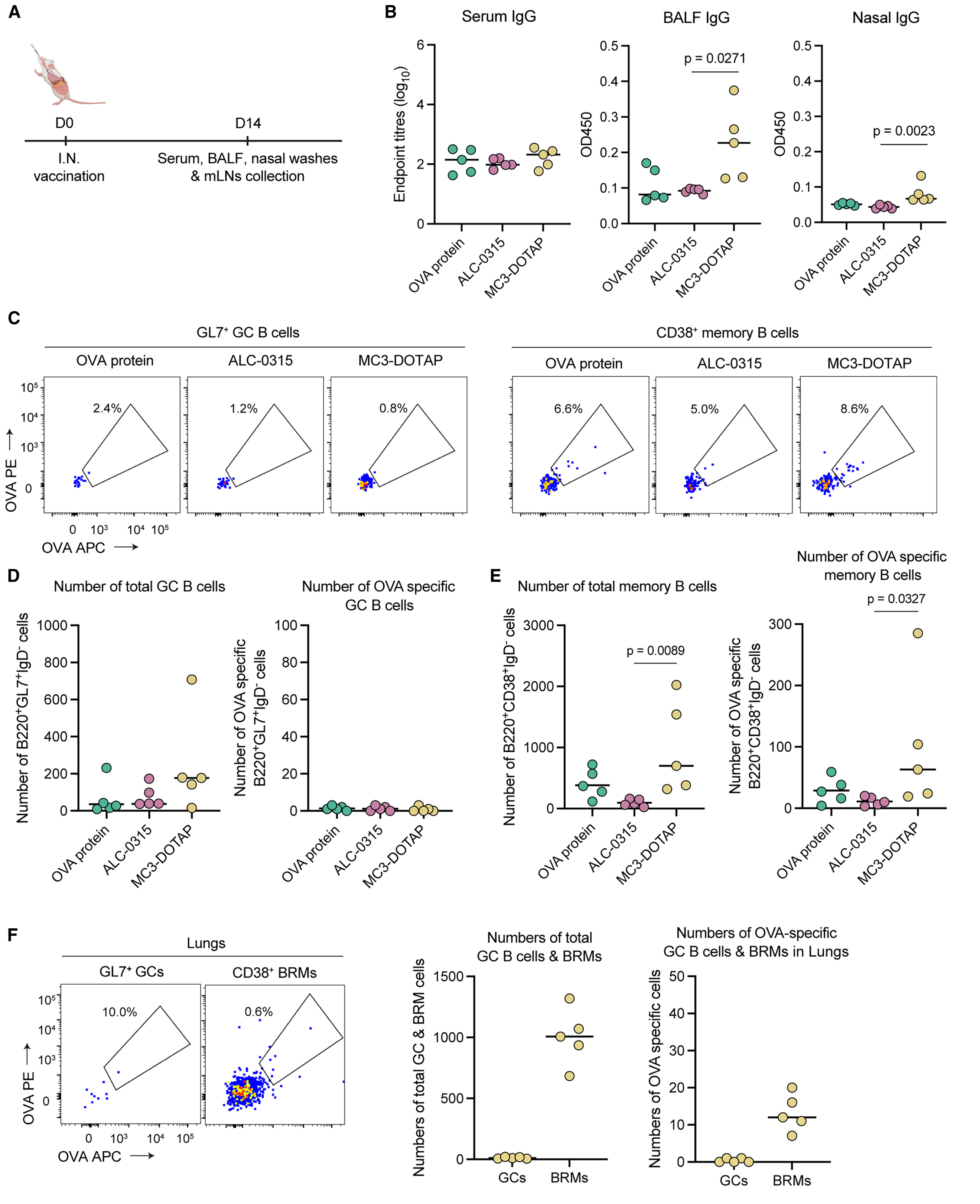

(legend on next page)

studies.[17] While evidence for direct biogenesis of immunity within the lung was limited, augmented antibody responses following i.n. mRNA boosting likely resulted from recalls of immune memory within the lung-draining mLNs. However, it remains unclear if this is driven by the drainage of vaccine antigens expressed in the lung or the drainage of mRNA-LNPs themselves. The incorporation of cationic lipid DOTAP in MC3 formulation appeared to enhance the recall responses when compared to the prototypic ALC-0315 mRNA-LNPs. One possible explanation for this could be the heightened transfection efficiency of MC3-DOTAP in airway epithelial cells although underlying mechanisms require further investigation.

In summary, we found that current mRNA-LNP formulations have limited utility for direct immunization at the respiratory mucosa, despite efficient cellular transfection. However, with the ability to recall B cell responses from distal lymphoid tissues, mRNA-LNPs might be promising candidates for use as i.n. boosters, with the addition of DOTAP potentially increasing the capacity to elicit recall. Efficient delivery of mRNA cargo to the mucosa also highlights potential utility for non-vaccine usage cases; for example, delivering mRNA therapies or antiviral monoclonal antibody therapeutics to the respiratory tract. Further optimization of mRNA-LNP formulations is likely required to balance mRNA delivery, immunogenicity, and safety at mucosal sites of administration.

MATERIALS AND METHODS

Ethics statement

Animal studies and related experimental procedures were approved by the University of Melbourne Animal Ethics Committee (ethics approval: 24909).

Preparation of mRNA LNPs

mRNA LNPs were prepared using the microfluidic mixing method.[19] Briefly, all lipids (DC Chemicals), including ionizable lipids (ALC-0315, SM-102, and DLin-MC3-DMA), cationic lipid (DOTAP), helper lipids (1,2-distearoyl-sn-glycero-3-phosphocholine [DSPC]), cholesterol, and PEG-lipids (ALC-0159 and DMG-PEG2000) with specific molecular ratios were dissolved in ethanol. DiD (Thermo Fisher Scientific) at 0.2 mol% of total lipid in the formulation was also dissolved in ethanol when applicable. OVA and CRE mRNA (TriLink BioTechnologies) were dissolved in acetate buffer (pH 4.0). The two solutions were mixed using microfluidic Ignite Cartridges on the NanoAssemblr Ignite (Precision NanoSystems) at an aqueous to ethanol ratio of 3/1 (vol/vol), a total flow rate of 8 mL min^{-1}, and a flow rate ratio of 3/1. The formulations were subsequently purified by dialyzing against 10% sucrose in Tris-buffered saline buffer (Sigma Aldrich) in 18–20 h at room temperature. The LNPs were then filtered (Millex 13 mm Durapore PVDF 0.45 μm; Merck) before storing at −80°C until use.

Characterization of mRNA LNPs

Dynamic Light Scattering (DLS): dynamic diameter and zeta potential of mRNA LNPs (50–100 μg mL^{-1} in PBS) were measured by DLS using a Malvern Zetasizer Nano Series. *Cryoelectron microscopy (cryo-EM):* sample preparation for cryo-EM was undertaken using a FEI Vitrobot Mark IV system. Briefly, copper grids (300-mesh) coated with lacey carbon film (ProSciTech) were glow-discharged, and then 4 μL of samples at 2 mg mL^{-1} were applied to grids within the Vitrobot apparatus. One blot step of 4 s was carried out with a blotting force of −1, with samples then immediately plunged into liquid ethane. The vitrified samples were then transferred into a Gatan 626 cryo-holder and imaged by a FEI Talos L120C cryo-TEM at a voltage of 120 kV and temperature at −175 to −170°C. Images were processed using a CETA 4×4k CMOS camera. *Encapsulation efficiency (EE):* the total amount of mRNA and unencapsulated mRNA in LNPs (with or without the addition of 1 μL of 10% Triton X-100 and continuous mixing at 300 rpm, 37°C in 8 min) were quantified using a Quant-iT RiboGreen RNA assay kit (Thermo Fisher Scientific) according to manufacture guidelines. The EE of mRNA in LNPs was calculated with the following formula: EE% = (total mRNA − unencapsulated mRNA) ÷ total mRNA × 100.

Rescue and propagation of viruses

Recombinant PR8 (A/Puerto Rico/8/1934) influenza viruses were rescued using eight-plasmid reverse genetics as previously described.[26] Genes encoding OVA immunogen were cloned into the NS-modified pHW200 vector. This along with plasmids encoding HA, NA, PA, NP, M, PB1, and PB2 were transfected into 293T cells. After overnight incubation, the 293T cells were removed and cocultured with MDCK cells for 72 h at 35°C. Viruses were plaque purified and propagated in MDCK cells to generate seed stocks which were used to inoculate 10-day-old embryonated chicken eggs. Eggs were incubated at 35°C for 3 days, chilled overnight, and allantoic fluid harvested. Rescued viruses were confirmed by hemagglutination assay and titrated by standard plaque assay.

Immunofluorescent assay

Immunofluorescent assay (IFA) was used to assess the expression of OVA and influenza HA protein from WT PR8 and OVA PR8 infection. Briefly, A549 (human lung epithelial) cells were inoculated with WT PR8 (MOI of 5) or OVA PR8 (MOI of 1). At 48 h post-incubation, the cells were fixed with 3.7% formaldehyde and permeabilized with 0.2% Triton X-100 for 15 min at room temperature. After that,

Figure 4. Effects of LNP formulation on humoral immune responses following intranasal vaccination of mRNA vaccines

(A) Groups of C57BL/6 (n = 5) were i.n. vaccinated with 2.0 μg OVA protein or OVA mRNA LNPs (2.0 μg mRNA). (B) OVA-specific IgG titers measured in mouse sera, BALF, and nasal washes at day 14 post vaccination using ELISA assays. (C) Representative flow plot of OVA-specific GC B cell populations ($IgD^{-}B220^{+}GL7^{+)}$ (left) and memory B cell populations ($IgD^{-}B220^{+}CD38^{+}$) (right) in mediastinal LNs at day 14 post vaccination. (D) Numbers of total and OVA-specific GC B cells. (E) Numbers of total and OVA-specific memory B cells. (F) Representative flow plot of OVA-specific GC B cells and tissue-resident memory B cells in lungs at day 14 post MC3-DOTAP i.n. vaccination (left) and numbers of total and OVA-specific GC and BRM cells in lungs (right). Statistical significance was determined by a Kruskal-Wallis test followed by post-hoc Dunn's multiple comparisons test.

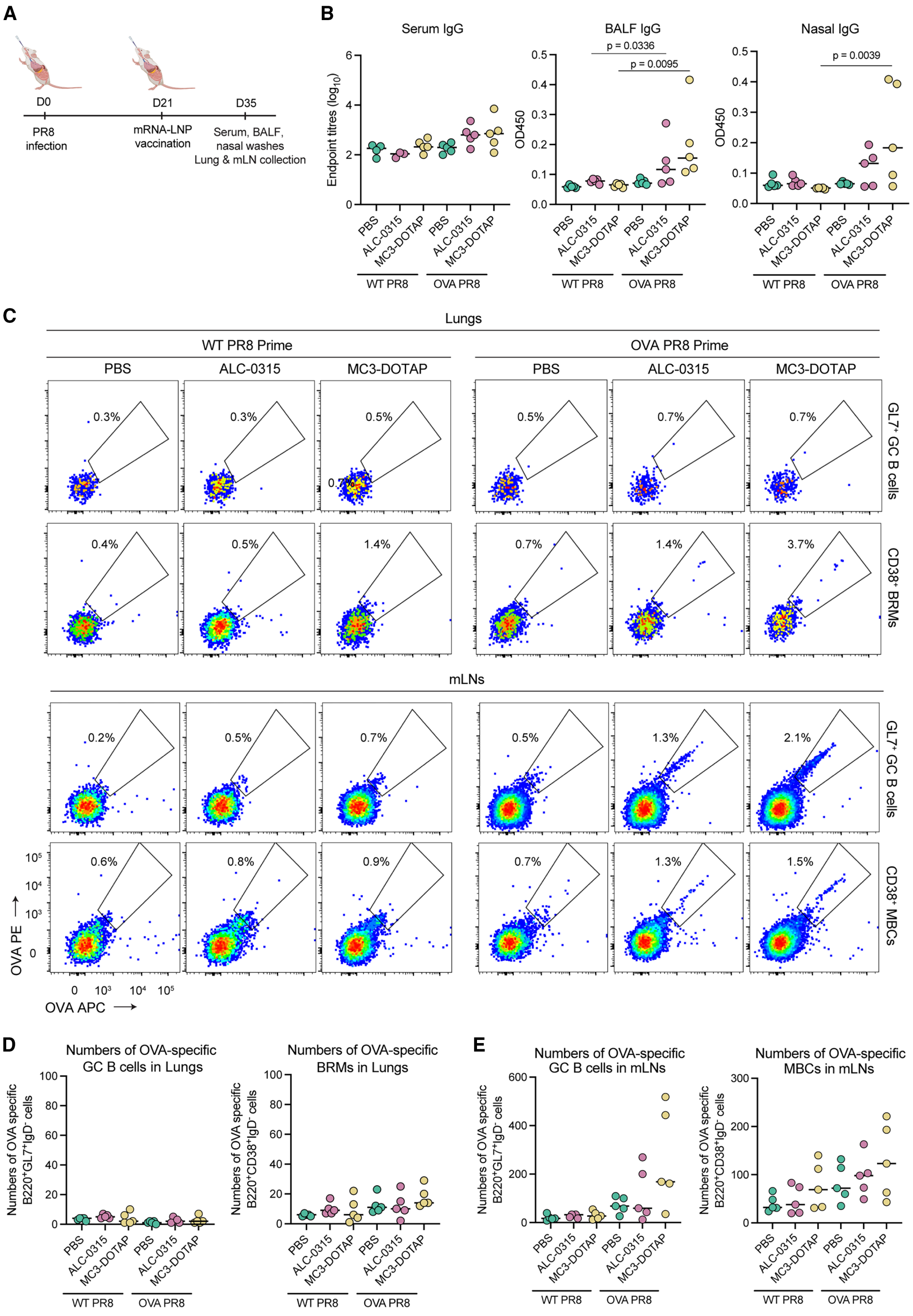

(legend on next page)

the cells were stained with mouse anti-OVA antibody (TOSGAAI, BioLegend) and human anti-HA antibody (TN1F11)[36] and then with secondary antibodies Alexa Fluor 488 goat anti-mouse IgG (H + L) and Alexa Fluor 647 goat anti-human IgG (H + L) (Invitrogen) to detect OVA and HA, respectively. The cells were observed under a fluorescent microscope.

Mouse immunization and infection

C57BL/6 female mice (6–12 weeks old, $n = 5$ per group) were used for all studies. Mice were anesthetized by isofluorane inhalation before vaccination. *Intramuscular injection:* mice were intramuscularly injected into hind quadriceps with either 5 μg OVA protein plus 50% volume of Addavax (InvivoGen) as adjuvants, or OVA mRNA LNPs at 5 μg mRNA. Mice were injected with 50 μL of vaccine solution in each leg. *Intranasal vaccination*: mice were intranasally administered with 50 μL of OVA protein at 2 μg or OVA mRNA LNPs at 2 μg mRNA. *Intranasal infection:* mice were intranasally administrated with 50 μL of either wild-type PR8 at 100 pfu or OVA PR8 at $10^{5.5}$ pfu. The mice were monitored for clinical signs and weight loss for 14 days post infection.

Collection of serum, BALF, nasal washes

At day 14 after each vaccination, blood, BALF, and nasal washes were collected. Blood samples were taken by submandibular or cardiac bleeds, and serum was isolated by centrifugation. To collect BALF and nasal washes, a small incision in the trachea was occupied to allow the insertion of a 20G cannula. Lungs were washed three times with 600 μL of PBS via the cannula to harvest BALF samples. Nasal washes were performed by washing the nasal cavity three times with 200 μL of PBS. All samples were stored at −80°C until use.

ELISA

Antigen-specific IgG titers in serum, BALF, and nasal washes collected from vaccinated mice were detected by a direct ELISA. The 96-well MaxiSorp plates were coated with 100 μL of OVA protein solution at 2 μg mL^{-1} in PBS overnight at 4°C. The plates were then blocked with 1% fetal calf serum (FCS) in PBS for 1 h at room temperature. All serum samples were diluted in blocking solution at 1:100, while only BALF samples from the i.m. prime - i.n. boost group were diluted at 1:10, followed by a serial 4-fold dilution. All other BALF samples and nasal washes were used as neat. The samples were added to the plates and incubated for 2 h at room temperature. The plates were next placed with anti-mouse IgG secondary antibodies which were conjugated with horseradish peroxidase (HRP) at 1:15,000 dilution in a blocking solution for 1 h at room temperature. The plates were then developed with 80 μL of 3,3′,5,5′-tetramethylbenzidine (TMB) in 8 min and stopped with 50 μL of 0.16 M sulfuric acid. Absorbance was measured at 450 nm. ELISA endpoint titers were calculated as the reciprocal of the sample dilution giving a signal 2× above background, while for neat samples, antibody levels were reported as optical density at 450 nm (OD450).

Flow cytometry detection of OVA-specific B cells

To delineate tissue-resident memory B cell populations in lungs, 10 min prior to culling, 2 μg of anti-CD45.2 (104, eBioscience) per mouse was administered intravenously. Mice were then sacrificed and dLNs and/or lungs were harvested in RF10 media (RPMI 1640, 10% FCS, 1× penicillin-streptomycin-glutamine). Single-cell suspensions were isolated by mechanical dissociation through a 70 μm cell strainer. Red blood cells from the lungs were lysed with PharmLyse (BD). Single cells were stained with Aqua viability dye (Thermo Fisher Scientific) and Fc-blocked with anti-CD16/CD32 antibodies. The cells were then labeled with OVA-PE and OVA-APC probes, and the following antibody panel: F4/80 BV786 (BM8; BioLegend), B220 BUV737 (RA3-6B2; BD), CD45 Cy7-APC (30-F11; BD), IgD BUV 395 (11-26c.2a; BD), GL7 AF488 (GL7; BioLegend), and CD38 Cy7-PE (clone 90; BioLegend). Cells were then fixed in 1% formaldehyde solution and analyzed on a BD LSR Fortessa using BD FACSDiva. Flow cytometry data were processed using FlowJo v10.

Flow cytometry detection of tdTom expression in immune cell subtypes in lungs

Ai14 mice (6–12 weeks old) were i.n. administrated with 2 μg CRE mRNA-LNPs. At 48 h post-administration, mice were sacrificed, and lungs were harvested and immediately fixed in 4% formaldehyde solution in 1 h at 4°C. The fixed lungs were then digested in collagenase D and DNase I solution for 30 min at 37°C. After that, single-cell suspensions were isolated by mechanical dissociation through a 70 μm cell strainer. Red blood cells from the lungs were lysed with PharmLyse (BD). Single cells were stained with Aqua viability dye (Thermo Fisher Scientific), and Fc-blocked with anti-CD16/CD32 antibodies. The cells were then labeled with following antibody panel: B220 BUV737 (RA3-6B2; BD), CD3e BV421 (145-2C11; BioLegend), F4/80 BV650 (BM8; BioLegend), CD11b BV711 (M1/70; BioLegend), CD11c BV785 (N418; BioLegend), Ly6G AF488 (AL-21; BD), CD103 PerCP-Cy5.5 (M290; BD), Ly6C AF700 (1A8, BioLegend), and CD45 Cy7-APC (30-F11; BD). Cells were then diluted in 1% FCS in PBS solution and analyzed on a BD LSR Fortessa using BD FACSDiva. Flow cytometry data were processed using FlowJo v10.

Confocal microscopy

Ai14 mice (6–12 weeks old) were either intramuscularly or intranasally administrated with CRE mRNA LNPs with or without DiD

Figure 5. Minimal B cell immunity established in pre-inflamed lungs following mRNA intranasal vaccination

(A) Groups of C57BL/6 ($n = 5$) were i.n. infected with either WT PR8 at 100 pfu or OVA PR8 at $10^{5.5}$ pfu. At day 21 post infection, mice were i.n. vaccinated with MC3-DOTAP, ALC-0315 OVA mRNA LNPs (2.0 μg mRNA) or PBS control. (B) OVA-specific IgG titers measured in mouse sera, BALF, and nasal washes at day 14 post boosting using ELISA assays. (C) Representative flow plot OVA-specific GC and memory B cells in lungs (top) and mLNs (bottom) at day 14 post i.n. boost. (D) Numbers of OVA-specific GC B cells and BRM cells in lungs. (E) Numbers of OVA-specific GC B cells and MBCs in mLNs at day 14 post i.n. boost. Statistical significance was determined by a Kruskal-Wallis test followed by post-hoc Dunn's multiple comparisons test.

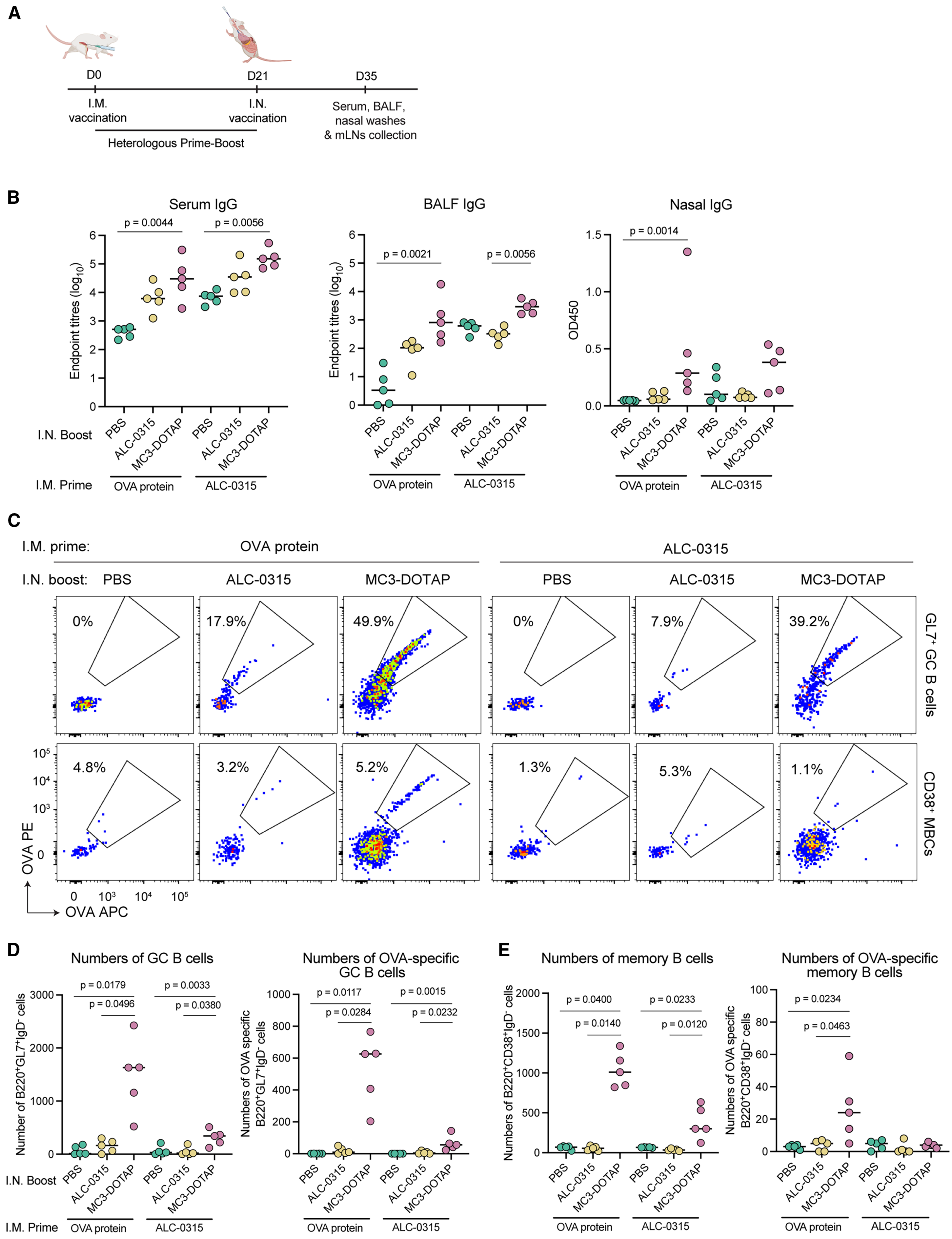

(legend on next page)

labeling at 5 μg or 2 μg mRNA, respectively. At 48 h post-administration, muscle and inguinal lymph nodes (i.m.) or lungs (i.n.) were harvested into 4% formaldehyde solution and fixed overnight at 4°C. The tissues were transferred into 30% sucrose solution to allow dehydration overnight at 4°C before embedding into OCT (Tissue-Tek), snap-frozen, and stored at − 80°C overnight. The tissues were then sectioned into 10 μm slices on a CryoStat (Leica) and dried overnight. Before staining, the sectioned tissues were rehydrated in PBS for 10 min and blocked with 5% bovine serum albumin (Millipore Sigma), 1:50 anti-CD16/CD32, and 2% (vol/vol) normal goat serum for 1 h at room temperature. The muscle was then stained with rabbit anti-Laminin (Sigma Aldrich) for 2 h and a cocktail of anti-rabbit AF488 (Life Technologies), CD45 AF647 (30-F11, BioLegend), and DAPI (Thermo Fisher Scientific) for another 2 h at room temperature. The lymph nodes were labeled with CD11c AF488 (N418, BioLegend) while lungs were stained with EpCAM AF488 (9C4, BioLegend) and CD45 AF647 (30-F11, BioLegend) for 2 h at room temperature. The slides were next mounted with Prolong Diamond Antifade Mountant (Life Technologies) and imaged by Zeiss LSM980 confocal microscopy using Zen Blue software. Images were captured with either a 10×, 20× air, or 60× oil objectives at 1 airy unit and a resolution of 512 × 512 or 1,024 × 1,024 pixels. Images were processed using FIJI or Imaris version 10 software. Percentage of tdTom expressed cells in lung sections was calculated by total tdTom positive cells divided by total DAPI positive cells. The Spots Close to Surface XTension in Imaris was used to determine the number of tdTom-expressed epithelial or immune cells which was the number of tdTom spots located inside a 10-threshold regions from EpCAM or CD45 staining surfaces, respectively.

Statistical analysis

Data were presented as median or median ±interquartile range (IQR) (n = 5). Statistical analyses were performed using GraphPad Prism version 10. For analyses comparing two groups, a non-parametric Mann-Whitney was performed. For other analyses comparing multiple groups, a Kruskal-Wallis test followed by post-hoc Dunn's multiple comparisons test was used. p values of less than 0.05 ($p < 0.05$) were considered to be significant for all statistical tests.

DATA AVAILABILITY

Any data is available upon request to the corresponding author.

ACKNOWLEDGMENTS

The graphical abstract and some portions of figures were created with BioRender (BioRender.com). The authors acknowledge the facilities and the scientific and technical assistance of the Bioresources Facility (BRF), Biological Optical Microscopy Platform (BOMP), Melbourne Cytometry Platform, and Ian Holmes Imaging Centre (IHIC) at the University of Melbourne. This work was supported by Australian Medical Research Future Fund grants 2005544 and 2013870, Australian National Health and Medical Research Council Investigator grants (M.K., S.J.K., J.A.J., H.-X.T., and A.K.W.), and University of Melbourne Early Career Researcher grant (M.N.V.).

AUTHOR CONTRIBUTIONS

M.N.V., H.-X.T., J.A.J., and A.K.W. designed the study. M.N.V. and D.P. performed experiments. A.K. synthesized and purified vaccine antigens. M.K. and S.J.K. provided guidance and discussion on experimental design. M.N.V., H.-X.T., J.A.J., and A.K.W. wrote the manuscript. All authors read and revised the manuscript.

DECLARATION OF INTERESTS

The authors declare no competing interests.

SUPPLEMENTAL INFORMATION

Supplemental information can be found online at https://doi.org/10.1016/j.omtn.2025.102547.

REFERENCES

1. Yusuf, H., and Kett, V. (2017). Current prospects and future challenges for nasal vaccine delivery. Hum. Vaccines Immunother. *13*, 34–45. https://doi.org/10.1080/21645515.2016.1239668.

2. Mouro, V., and Fischer, A. (2022). Dealing with a mucosal viral pandemic: lessons from COVID-19 vaccines. Mucosal Immunol. *15*, 584–594. https://doi.org/10.1038/s41385-022-00517-8.

3. Sterlin, D., Mathian, A., Miyara, M., Mohr, A., Anna, F., Claër, L., Quentric, P., Fadlallah, J., Devilliers, H., Ghillani, P., et al. (2021). IgA dominates the early neutralizing antibody response to SARS-CoV-2. Sci. Transl. Med. *13*, eabd2223. https://doi.org/10.1126/scitranslmed.abd2223.

4. Afkhami, S., D'Agostino, M.R., Zhang, A., Stacey, H.D., Marzok, A., Kang, A., Singh, R., Bavananthasivam, J., Ye, G., Luo, X., et al. (2022). Respiratory mucosal delivery of next-generation COVID-19 vaccine provides robust protection against both ancestral and variant strains of SARS-CoV-2. Cell *185*, 896–915.e19. https://doi.org/10.1016/j.cell.2022.02.005.

5. Lei, H., Alu, A., Yang, J., Ren, W., He, C., Lan, T., He, X., Yang, L., Li, J., Wang, Z., et al. (2022). Intranasal administration of a recombinant RBD vaccine induces long-term immunity against Omicron-included SARS-CoV-2 variants. Signal Transduct. Target. Ther. *7*, 159. https://doi.org/10.1038/s41392-022-01002-1.

6. Kissler, S.M., Fauver, J.R., Mack, C., Tai, C.G., Breban, M.I., Watkins, A.E., Samant, R.M., Anderson, D.J., Metti, J., Khullar, G., et al. (2021). Viral Dynamics of SARS-CoV-2 Variants in Vaccinated and Unvaccinated Persons. N. Engl. J. Med. *385*, 2489–2491. https://doi.org/10.1056/NEJMc2102507.

7. Mao, T., Israelow, B., Peña-Hernández, M.A., Suberi, A., Zhou, L., Luyten, S., Reschke, M., Dong, H., Homer, R.J., Saltzman, W.M., and Iwasaki, A. (2022). Unadjuvanted intranasal spike vaccine elicits protective mucosal immunity against sarbecoviruses. Science *378*, eabo2523. https://doi.org/10.1126/science.abo2523.

8. Pilapitiya, D., Wheatley, A.K., and Tan, H.-X. (2023). Mucosal vaccines for SARS-CoV-2: triumph of hope over experience. EBioMedicine *92*, 104585. https://doi.org/10.1016/j.ebiom.2023.104585.

9. Madhavan, M., Ritchie, A.J., Aboagye, J., Jenkin, D., Provstgaad-Morys, S., Tarbet, I., Woods, D., Davies, S., Baker, M., Platt, A., et al. (2022). Tolerability and immunogenicity of an intranasally administered adenovirus-vectored COVID-19 vaccine:

Figure 6. Augmented systemic and mucosal humoral immune responses following intranasal boosting with MC3-DOTAP in mice with pre-existing antigen-specific systemic immunity

(A) Groups of C57BL/6 (n = 5) were i.m. primed with either 5 μg OVA protein plus 50% volume of Addavax or ALC-0315 OVA mRNA LNPs (5 μg mRNA), and then i.n. boosted with either ALC-0315, MC3-DOTAP OVA mRNA LNPs (2.0 μg mRNA) or PBS control. (B) OVA-specific IgG titers measured in mouse sera, BALF, and nasal washes at day 14 post boost using ELISA assays. (C) Representative flow plot of OVA-specific GC and memory B cell populations in mLNs at day 14 post boosting. (D) Numbers of total and OVA-specific GC B cells. (E) Numbers of total and OVA-specific memory B cells in mLNs. Statistical significance was determined by a Kruskal-Wallis test followed by post-hoc Dunn's multiple comparisons test.

An open-label partially-randomised ascending dose phase I trial. EBioMedicine *85*, 104298. https://doi.org/10.1016/j.ebiom.2022.104298.

10. Wu, S., Huang, J., Zhang, Z., Wu, J., Zhang, J., Hu, H., Zhu, T., Zhang, J., Luo, L., Fan, P., et al. (2021). Safety, tolerability, and immunogenicity of an aerosolised adenovirus type-5 vector-based COVID-19 vaccine (Ad5-nCoV) in adults: preliminary report of an open-label and randomised phase 1 clinical trial. Lancet Infect. Dis. *21*, 1654–1664. https://doi.org/10.1016/S1473-3099(21)00396-0.
11. Kirtane, A.R., Tang, C., Freitas, D., Bernstock, J.D., and Traverso, G. (2023). Challenges and opportunities in the development of mucosal mRNA vaccines. Curr. Opin. Immunol. *85*, 102388. https://doi.org/10.1016/j.coi.2023.102388.
12. (2021). Let's talk about lipid nanoparticles. Nat. Rev. Mater. *6*, 99. https://doi.org/10.1038/s41578-021-00281-4.
13. Alameh, M.G., Tombácz, I., Bettini, E., Lederer, K., Sittplangkoon, C., Wilmore, J.R., Gaudette, B.T., Soliman, O.Y., Pine, M., Hicks, P., et al. (2021). Lipid nanoparticles enhance the efficacy of mRNA and protein subunit vaccines by inducing robust T follicular helper cell and humoral responses. Immunity *54*, 2877–2892.e7. https://doi.org/10.1016/j.immuni.2021.11.001.
14. McMahan, K., Wegmann, F., Aid, M., Sciacca, M., Liu, J., Hachmann, N.P., Miller, J., Jacob-Dolan, C., Powers, O., Hope, D., et al. (2024). Mucosal boosting enhances vaccine protection against SARS-CoV-2 in macaques. Nature *626*, 385–391. https://doi.org/10.1038/s41586-023-06951-3.
15. Lei, H., Alu, A., Yang, J., He, C., Hong, W., Cheng, Z., Yang, L., Li, J., Wang, Z., Wang, W., et al. (2020). Cationic nanocarriers as potent adjuvants for recombinant S-RBD vaccine of SARS-CoV-2. Signal Transduct. Target. Ther. *5*, 291. https://doi.org/10.1038/s41392-020-00434-x.
16. Baldeon Vaca, G., Meyer, M., Cadete, A., Hsiao, C.J., Golding, A., Jeon, A., Jacquinet, E., Azcue, E., Guan, C.M., Sanchez-Felix, X., et al. (2023). Intranasal mRNA-LNP vaccination protects hamsters from SARS-CoV-2 infection. Sci. Adv. *9*, eadh1655. https://doi.org/10.1126/sciadv.adh1655.
17. Jennewein, M.F., Schultz, M.D., Beaver, S., Battisti, P., Bakken, J., Hanson, D., Akther, J., Mohamath, R., Singh, J., Cross, N., et al. (2023). Intranasal self-amplifying RNA SARS-CoV-2 vaccine produces protective respiratory and systemic immunity and prevents viral transmission. Preprint at bioRxiv. https://doi.org/10.1101/2022.11.10.515993.
18. Cheng, Q., Wei, T., Farbiak, L., Johnson, L.T., Dilliard, S.A., and Siegwart, D.J. (2020). Selective organ targeting (SORT) nanoparticles for tissue-specific mRNA delivery and CRISPR–Cas gene editing. Nat. Nanotechnol. *15*, 313–320. https://doi.org/10.1038/s41565-020-0669-6.
19. Algarni, A., Pilkington, E.H., Suys, E.J.A., Al-Wassiti, H., Pouton, C.W., and Truong, N.P. (2022). In vivo delivery of plasmid DNA by lipid nanoparticles: the influence of ionizable cationic lipids on organ-selective gene expression. Biomater. Sci. *10*, 2940–2952. https://doi.org/10.1039/D2BM00168C.
20. Victora, G.D., and Nussenzweig, M.C. (2022). Germinal Centers. Annu. Rev. Immunol. *40*, 413–442. https://doi.org/10.1146/annurev-immunol-120419-022408.
21. Kauffman, K.J., Oberli, M.A., Dorkin, J.R., Hurtado, J.E., Kaczmarek, J.C., Bhadani, S., Wyckoff, J., Langer, R., Jaklenec, A., and Anderson, D.G. (2018). Rapid, Single-Cell Analysis and Discovery of Vectored mRNA Transfection In Vivo with a loxP-Flanked tdTomato Reporter Mouse. Mol. Ther. Nucleic Acids *10*, 55–63. https://doi.org/10.1016/j.omtn.2017.11.005.
22. Hwang, J.Y., Randall, T.D., and Silva-Sanchez, A. (2016). Inducible Bronchus-Associated Lymphoid Tissue: Taming Inflammation in the Lung. Front. Immunol. *7*, 258. https://doi.org/10.3389/fimmu.2016.00258.
23. Tan, H.-X., Esterbauer, R., Vanderven, H.A., Juno, J.A., Kent, S.J., and Wheatley, A.K. (2019). Inducible Bronchus-Associated Lymphoid Tissues (iBALT) Serve as Sites of B Cell Selection and Maturation Following Influenza Infection in Mice. Front. Immunol. *10*, 611. https://doi.org/10.3389/fimmu.2019.00611.
24. Tan, H.X., Juno, J.A., Esterbauer, R., Kelly, H.G., Wragg, K.M., Konstandopoulos, P., Alcantara, S., Alvarado, C., Jones, R., Starkey, G., et al. (2022). Lung-resident memory B cells established after pulmonary influenza infection display distinct transcriptional and phenotypic profiles. Sci. Immunol. *7*, eabf5314. https://doi.org/10.1126/sciimmunol.abf5314.
25. Poon, M.M.L., Rybkina, K., Kato, Y., Kubota, M., Matsumoto, R., Bloom, N.I., Zhang, Z., Hastie, K.M., Grifoni, A., Weiskopf, D., et al. (2021). SARS-CoV-2 infection generates tissue-localized immunological memory in humans. Sci. Immunol. *6*, eabl9105. https://doi.org/10.1126/sciimmunol.abl9105.
26. Hoffmann, E., Neumann, G., Kawaoka, Y., Hobom, G., and Webster, R.G. (2000). A DNA transfection system for generation of influenza A virus from eight plasmids. Proc. Natl. Acad. Sci. USA *97*, 6108–6113. https://doi.org/10.1073/pnas.100133697.
27. Tan, H.-X., Gilbertson, B.P., Jegaskanda, S., Alcantara, S., Amarasena, T., Stambas, J., McAuley, J.L., Kent, S.J., and De Rose, R. (2016). Recombinant influenza virus expressing HIV-1 p24 capsid protein induces mucosal HIV-specific CD8 T-cell responses. Vaccine *34*, 1172–1179. https://doi.org/10.1016/j.vaccine.2016.01.030.
28. Pilapitiya, D., Lee, W.S., Vu, M.N., Kelly, A., Webster, R.H., Koutsakos, M., Kent, S.J., Juno, J.A., Tan, H.-X., and Wheatley, A.K. (2025). Mucosal vaccination against SARS-CoV-2 using recombinant influenza viruses delivering self-assembling nanoparticles. Vaccine *46*, 126668. https://doi.org/10.1016/j.vaccine.2024.126668.
29. Ndeupen, S., Qin, Z., Jacobsen, S., Bouteau, A., Estanbouli, H., and Igyártó, B.Z. (2021). The mRNA-LNP platform's lipid nanoparticle component used in preclinical vaccine studies is highly inflammatory. iScience *24*, 103479. https://doi.org/10.1016/j.isci.2021.103479.
30. Moghimi, S.M., and Simberg, D. (2022). Pro-inflammatory concerns with lipid nanoparticles. Mol. Ther. *30*, 2109–2110. https://doi.org/10.1016/j.ymthe.2022.04.011.
31. Fifis, T., Gamvrellis, A., Crimeen-Irwin, B., Pietersz, G.A., Li, J., Mottram, P.L., McKenzie, I.F.C., and Plebanski, M. (2004). Size-Dependent Immunogenicity: Therapeutic and Protective Properties of Nano-Vaccines against Tumors. J. Immunol. *173*, 3148–3154. https://doi.org/10.4049/jimmunol.173.5.3148.
32. Hassett, K.J., Benenato, K.E., Jacquinet, E., Lee, A., Woods, A., Yuzhakov, O., Himansu, S., Deterling, J., Geilich, B.M., Ketova, T., et al. (2019). Optimization of Lipid Nanoparticles for Intramuscular Administration of mRNA Vaccines. Mol. Ther. Nucleic Acids *15*, 1–11. https://doi.org/10.1016/j.omtn.2019.01.013.
33. Hassan, A.O., Kafai, N.M., Dmitriev, I.P., Fox, J.M., Smith, B.K., Harvey, I.B., Chen, R.E., Winkler, E.S., Wessel, A.W., Case, J.B., et al. (2020). A Single-Dose Intranasal ChAd Vaccine Protects Upper and Lower Respiratory Tracts against SARS-CoV-2. Cell *183*, 169–184.e13. https://doi.org/10.1016/j.cell.2020.08.026.
34. Cokarić Brdovčak, M., Materljan, J., Šustić, M., Ravlić, S., Ružić, T., Lisnić, B., Miklić, K., Brizić, I., Pribanić Matešić, M., Juranić Lisnić, V., et al. (2022). ChAdOx1-S adenoviral vector vaccine applied intranasally elicits superior mucosal immunity compared to the intramuscular route of vaccination. Eur. J. Immunol. *52*, 936–945. https://doi.org/10.1002/eji.202249823.
35. van Doremalen, N., Purushotham, J.N., Schulz, J.E., Holbrook, M.G., Bushmaker, T., Carmody, A., Port, J.R., Yinda, C.K., Okumura, A., Saturday, G., et al. (2021). Intranasal ChAdOx1 nCoV-19/AZD1222 vaccination reduces viral shedding after SARS-CoV-2 D614G challenge in preclinical models. Sci. Transl. Med. *13*, eabh0755. https://doi.org/10.1126/scitranslmed.abh0755.
36. Kelly, H.G., Tan, H.-X., Juno, J.A., Esterbauer, R., Ju, Y., Jiang, W., Wimmer, V.C., Duckworth, B.C., Groom, J.R., Caruso, F., et al. (2020). Self-assembling influenza nanoparticle vaccines drive extended germinal center activity and memory B cell maturation. JCI Insight *5*, e136653. https://doi.org/10.1172/jci.insight.136653.

OMTN, Volume *36*

Supplemental information

Deconvolution of cargo delivery

and immunogenicity following intranasal

delivery of mRNA lipid nanoparticle vaccines

Mai N. Vu, Devaki Pilapitiya, Andrew Kelly, Marios Koutsakos, Stephen J. Kent, Jennifer A. Juno, Hyon-Xhi Tan, and Adam K. Wheatley

SUPPLEMENTAL MATERIALS

a

Cationic Lipid	Ionizable Lipid	Helper Lipid	Cholesterol	PEG-Lipid	Lipid ratio*	N:P ratio
- DOTAP	ALC-0315	DSPC	Cholesterol	ALC-0159	46.3/9.4/42.7/1.6	1:5
	SM-102	DSPC	Cholesterol	DMG-PEG2000	50.0/10.0/38.5/1.5	1:5
	Dlin-MC3-DMA	DSPC	Cholesterol	DSPE-PEG2000	52.0/8.0/38.5/1.5	1:5
+ DOTAP (50%)	SM-102	DSPC	Cholesterol	DMG-PEG2000	25.0/5.0/19.2/0.8	1:6
	Dlin-MC3-DMA	DSPC	Cholesterol	DSPE-PEG2000	25.0/5.0/19.2/0.8	1:6

*Ionizable lipid/ Helper lipid/ Cholesterol/ PEG-lipid mole ratio

b

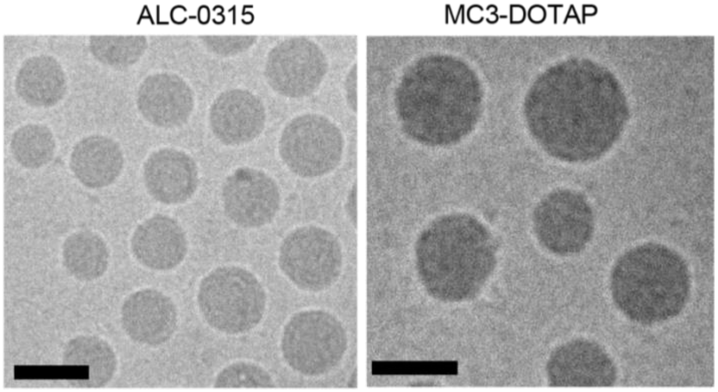

Figure S1: **a**). Formulations of mRNA LNPs. **b**). Representative cryoEM images of ALC-0315 and MC3-DOTAP OVA mRNA LNPs, scale bars = 50 nm.

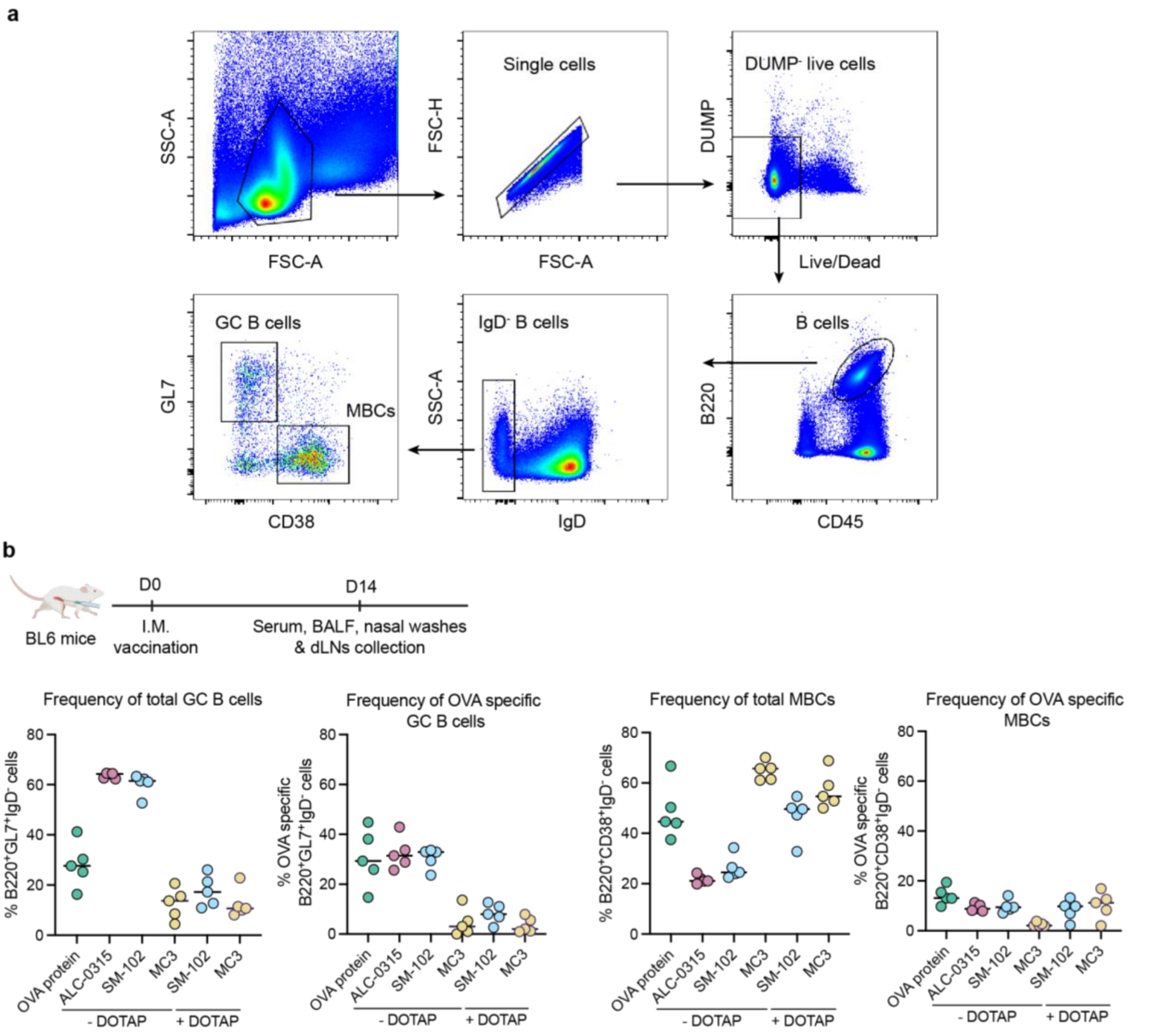

Figure S2: **a**). Gating strategies to identify GC B cells (B220+IgD−GL7+) and memory B cells (B220+IgD−CD38+) in lymph nodes and lungs. **b**). Frequency of total and OVA-specific GC B cells and memory B cells in dLNs at day 14 post i.m. vaccination of OVA protein and OVA mRNA-LNPs (related to figure 1).

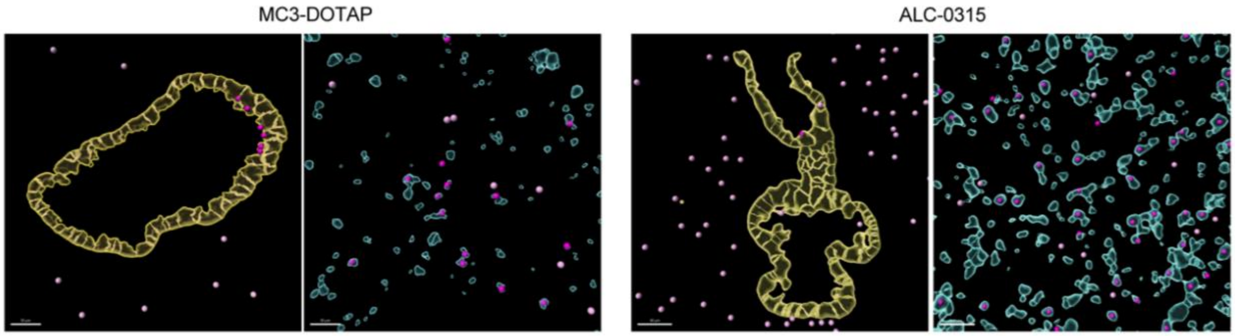

Figure S3: The Spots Close to Surface XTension in Imaris was used to determine the number of tdTom-expressed epithelial (yellow) or immune (cyan) cells. The cells with tdTom spots located inside a 10-thredshold regions (dark magenta) from EpCAM or CD45 staining surfaces were considered tdTom positive cells. In contrast, the cells with tdTom spots located outside the 10-thredshold regions (light magenta) were considered tdTom negative cells.

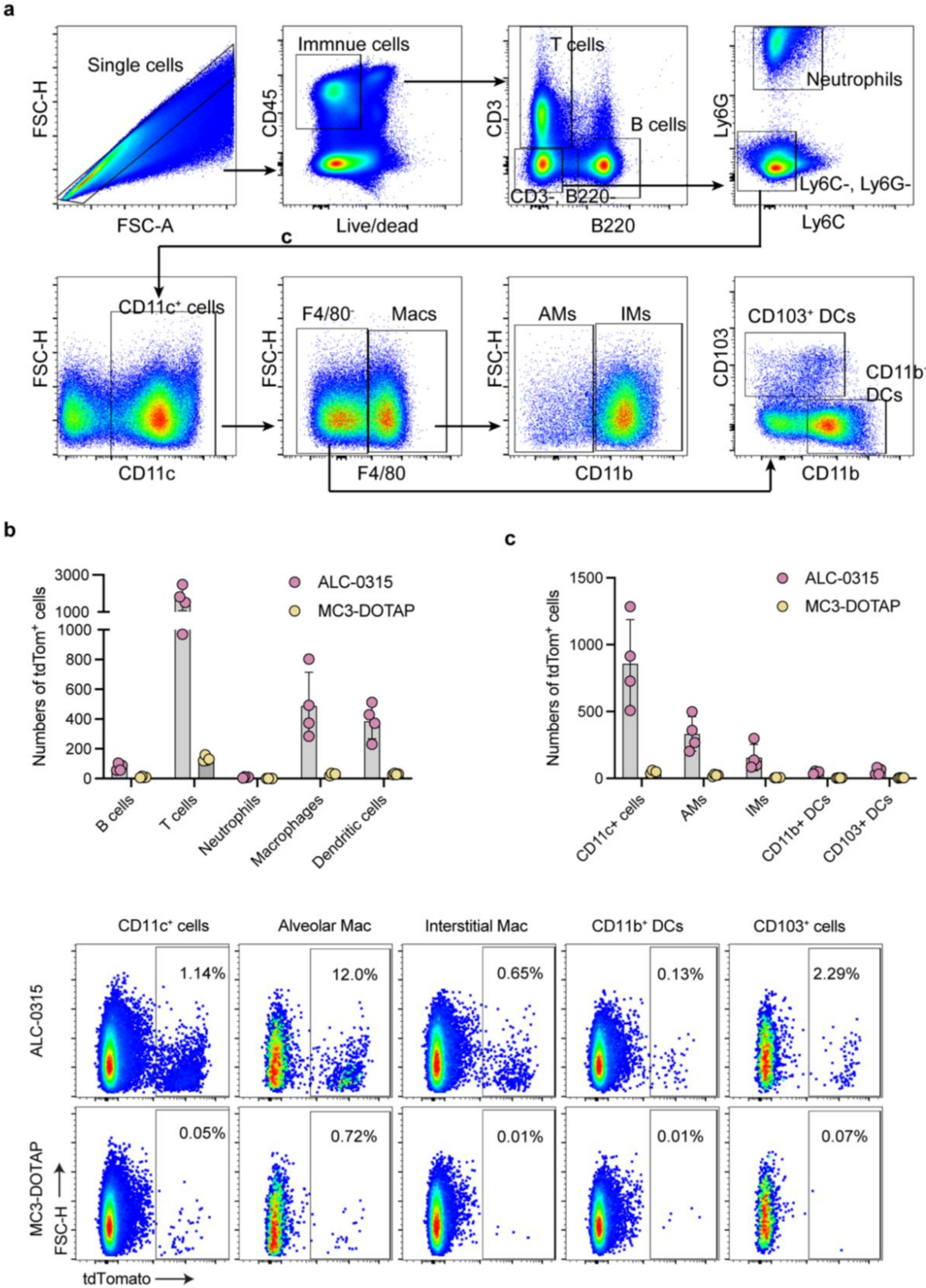
a
Single cells
FSC-H
FSC-A
Immnue cells
CD45
Live/dead
T cells
B cells
CD3
CD3-, B220-
B220
Neutrophils
Ly6G
Ly6C-, Ly6G-
Ly6C
c
CD11c⁺ cells
CD11c
F4/80⁻
Macs
F4/80
AMs
IMs
CD11b
CD103⁺ DCs
CD11b⁺ DCs
CD103
b
Numbers of tdTom⁺ cells
ALC-0315
MC3-DOTAP
B cells
T cells
Neutrophils
Macrophages
Dendritic cells
c
CD11c+ cells
AMs
IMs
CD11b+ DCs
CD103+ DCs
CD11c⁺ cells
Alveolar Mac
Interstitial Mac
CD11b⁺ DCs
CD103⁺ cells
1.14%
12.0%
0.65%
0.13%
2.29%
0.05%
0.72%
0.01%
0.01%
0.07%
FSC-H
tdTomato

Figure S4: **a**). Gating strategies to identify subtypes of immune cells in lungs following CRE mRNA-LNP intranasal vaccination, including CD45+ immune cells, CD3+ T cells, B220+ B cells, Ly6G+ neutrophils, CD11c+ F4/80+ macrophages with CD11b+ interstitial macrophages (IMs) and CD11b- alveolar macrophages (AMs), CD11c+ F4/80- dendritic cells (DCs) with CD11b+ DCs and DC103+ DCs. **b**). Numbers of tdTom positive cells in different CD45+ immune cell subsets in lungs. **c**). Numbers of tdTom positive cells in different dendritic and macrophage cell subtypes (top) and representative images showing tdTom positive cell populations in different dendritic and macrophage cell subsets in lungs (bottom).

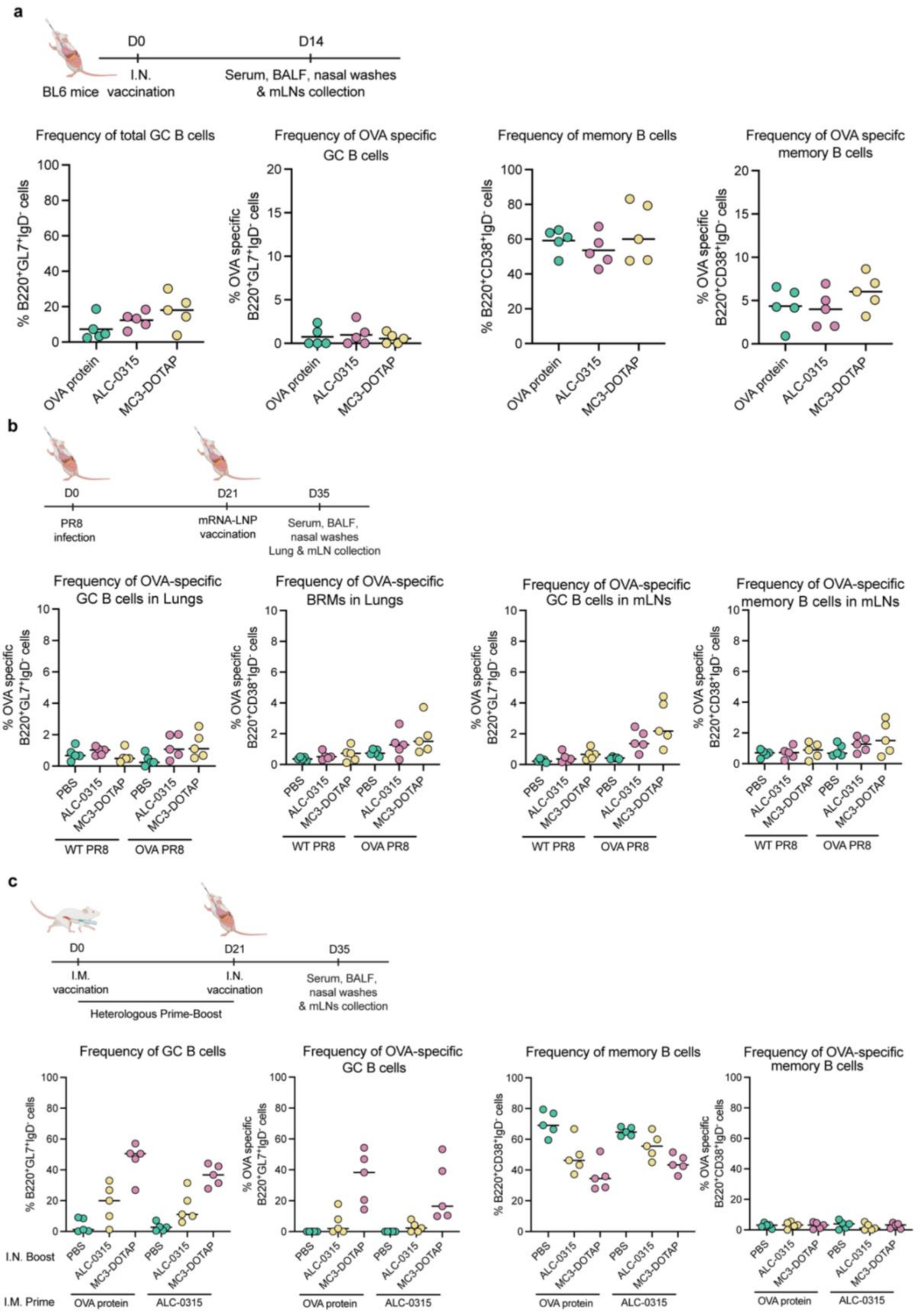
a
D0
D14
I.N. vaccination
Serum, BALF, nasal washes & mLNs collection
BL6 mice
Frequency of total GC B cells
% B220⁺GL7⁺IgD⁻ cells
Frequency of OVA specific GC B cells
% OVA specific B220⁺GL7⁺IgD⁻ cells
Frequency of memory B cells
% B220⁺CD38⁺IgD⁻ cells
Frequency of OVA specifc memory B cells
% OVA specific B220⁺CD38⁺IgD⁻ cells
OVA protein
ALC-0315
MC3-DOTAP
b
D0
D21
D35
PR8 infection
mRNA-LNP vaccination
Serum, BALF, nasal washes Lung & mLN collection
Frequency of OVA-specific GC B cells in Lungs
Frequency of OVA-specific BRMs in Lungs
Frequency of OVA-specific GC B cells in mLNs
Frequency of OVA-specific memory B cells in mLNs
% OVA specific B220⁺GL7⁺IgD⁻ cells
% OVA specific B220⁺CD38⁺IgD⁻ cells
PBS
ALC-0315
MC3-DOTAP
WT PR8
OVA PR8
c
D0
D21
D35
I.M. vaccination
I.N. vaccination
Heterologous Prime-Boost
Serum, BALF, nasal washes & mLNs collection
Frequency of GC B cells
% B220⁺GL7⁺IgD⁻ cells
Frequency of OVA-specific GC B cells
% OVA specific B220⁺GL7⁺IgD⁻ cells
Frequency of memory B cells
% B220⁺CD38⁺IgD⁻ cells
Frequency of OVA-specific memory B cells
% OVA specific B220⁺CD38⁺IgD⁻ cells
I.N. Boost
I.M. Prime
PBS
ALC-0315
MC3-DOTAP
OVA protein
ALC-0315

Figure S5: Frequencies of GC B cells and memory B cells after mRNA-LNP vaccination. **a**). Groups of C57BL/6 (n = 5) were i.n. vaccinated with 2.0 μg OVA protein or OVA mRNA LNPs at 2.0 μg mRNA (top) and frequencies of total and OVA-specific GC B cells and MBCs in mLNs at day 14 post vaccination (bottom) (related to figure 4). **b**). Groups of C57BL/6 (n = 5) were i.n. infected with either WT PR8 at 100 pfu or OVA PR8 at $10^{5.5}$ pfu. At day 21 post infection, mice were i.n. vaccinated with MC3-DOTAP, ALC-0315 OVA mRNA LNPs at 2.0 μg mRNA or PBS control (top) and frequencies of OVA-specific GC B cells and memory B cells in lungs and mLNs at day 14 post boost (bottom) (related to figure 5). **c**). Groups of C57BL/6 (n = 5) were i.m. primed with either 5 μg OVA protein plus 50% volume of Addavax, or ALC-0315 OVA mRNA LNPs at 5 μg mRNA and then i.n. boosted with either ALC-0315, MC3-DOTAP OVA mRNA LNPs at 2.0 μg or PBS control (top) and frequencies of total and OVA-specific GC B cells and MBCs in mLNs at day 14 post boost (bottom) (related to Fig. 6).

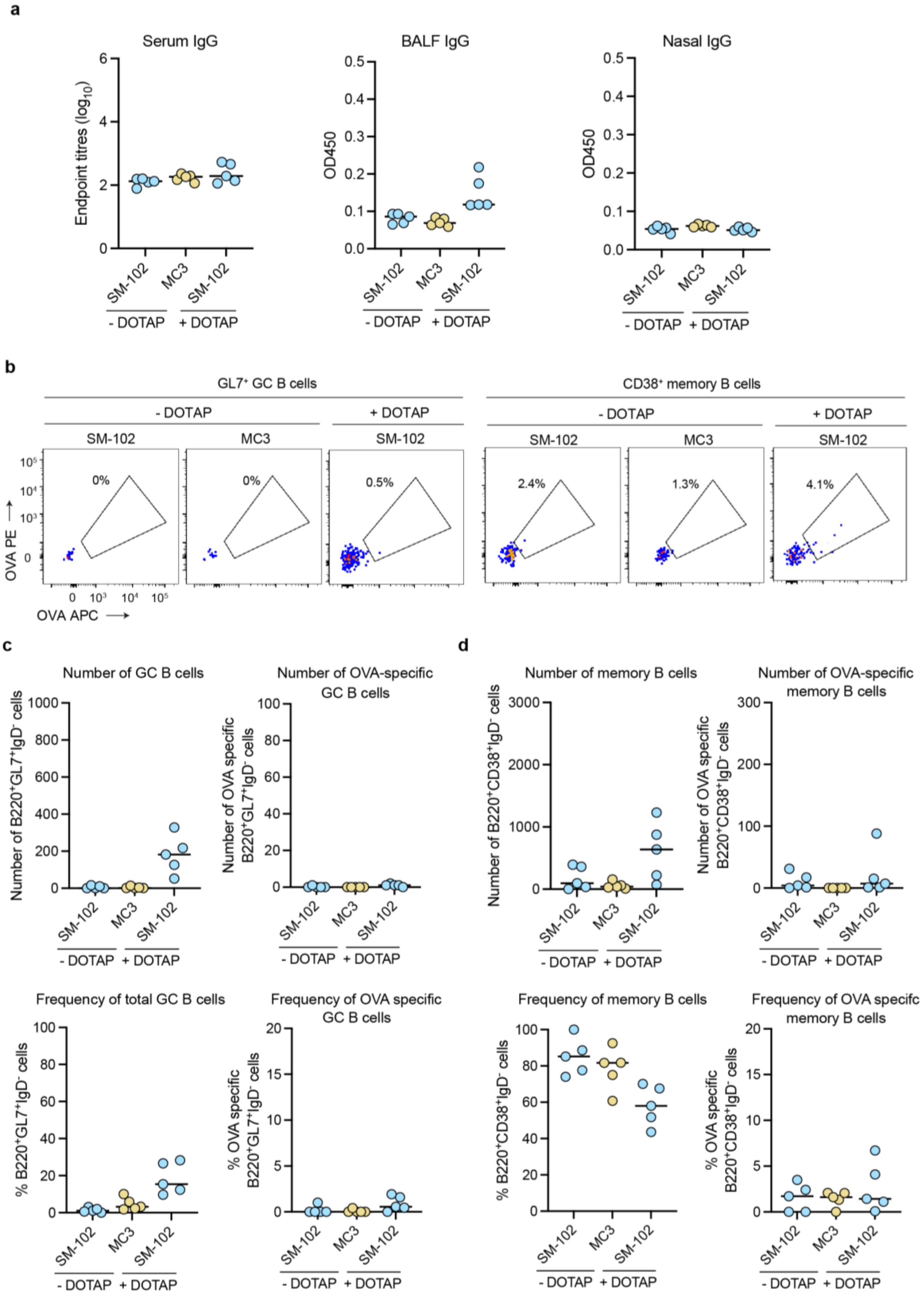
a
Serum IgG
Endpoint titres (log_{10})
BALF IgG
OD450
Nasal IgG
OD450
SM-102
MC3
SM-102
- DOTAP
+ DOTAP
b
GL7⁺ GC B cells
CD38⁺ memory B cells
- DOTAP
+ DOTAP
SM-102
MC3
SM-102
0%
0%
0.5%
2.4%
1.3%
4.1%
OVA PE
OVA APC
c
Number of GC B cells
Number of B220⁺GL7⁺IgD⁻ cells
Number of OVA-specific GC B cells
Number of OVA specific B220⁺GL7⁺IgD⁻ cells
Frequency of total GC B cells
% B220⁺GL7⁺IgD⁻ cells
Frequency of OVA specific GC B cells
% OVA specific B220⁺GL7⁺IgD⁻ cells
d
Number of memory B cells
Number of B220⁺CD38⁺IgD⁻ cells
Number of OVA-specific memory B cells
Number of OVA specific B220⁺CD38⁺IgD⁻ cells
Frequency of memory B cells
% B220⁺CD38⁺IgD⁻ cells
Frequency of OVA specifc memory B cells
% OVA specific B220⁺CD38⁺IgD⁻ cells

Figure S6: Systemic and mucosal antibody responses to SM-102, MC3, and SM-102-DOTAP OVA mRNA LNPs at 14 days post i.n. vaccination. Groups of C57BL/6 (n = 5) were i.n. vaccinated with the mRNA LNPs at 2.0 μg mRNA. **a**). OVA-specific IgG titers measured in mouse sera, BALF, and nasal washes using ELISA assays. **b**). Representative flow plot of OVA-specific GC and memory B cell populations in mLNs. **c**). Numbers and frequencies of total and OVA-specific GC B cells. **d**). Numbers and frequencies of total and OVA-specific memory B cells.

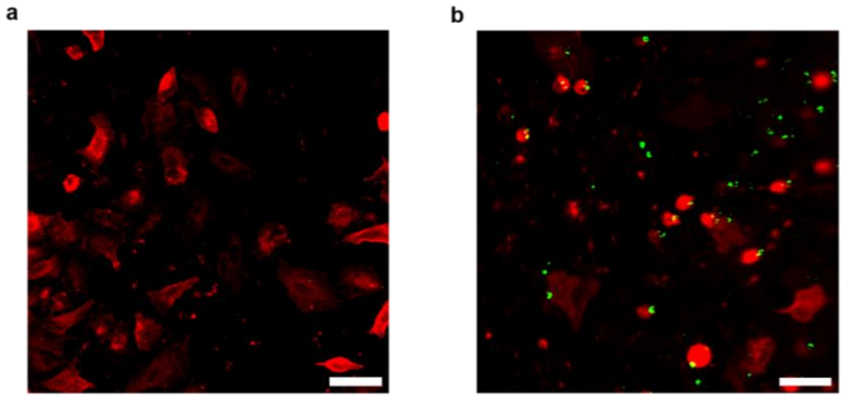

Figure S7: Expression of influenza hemagglutinin (HA; red) or ovalbumin (OVA; green) was assessed in A549 cells at 48h post-infection using immunofluorescent microscopy. **a**). Cells infected with A/Puerto Rico/08/1934 virus alone (MOI of 5). **b**). Cells infected with PR8 expressing OVA (MOI of 1). Scale bars = 100 μm.

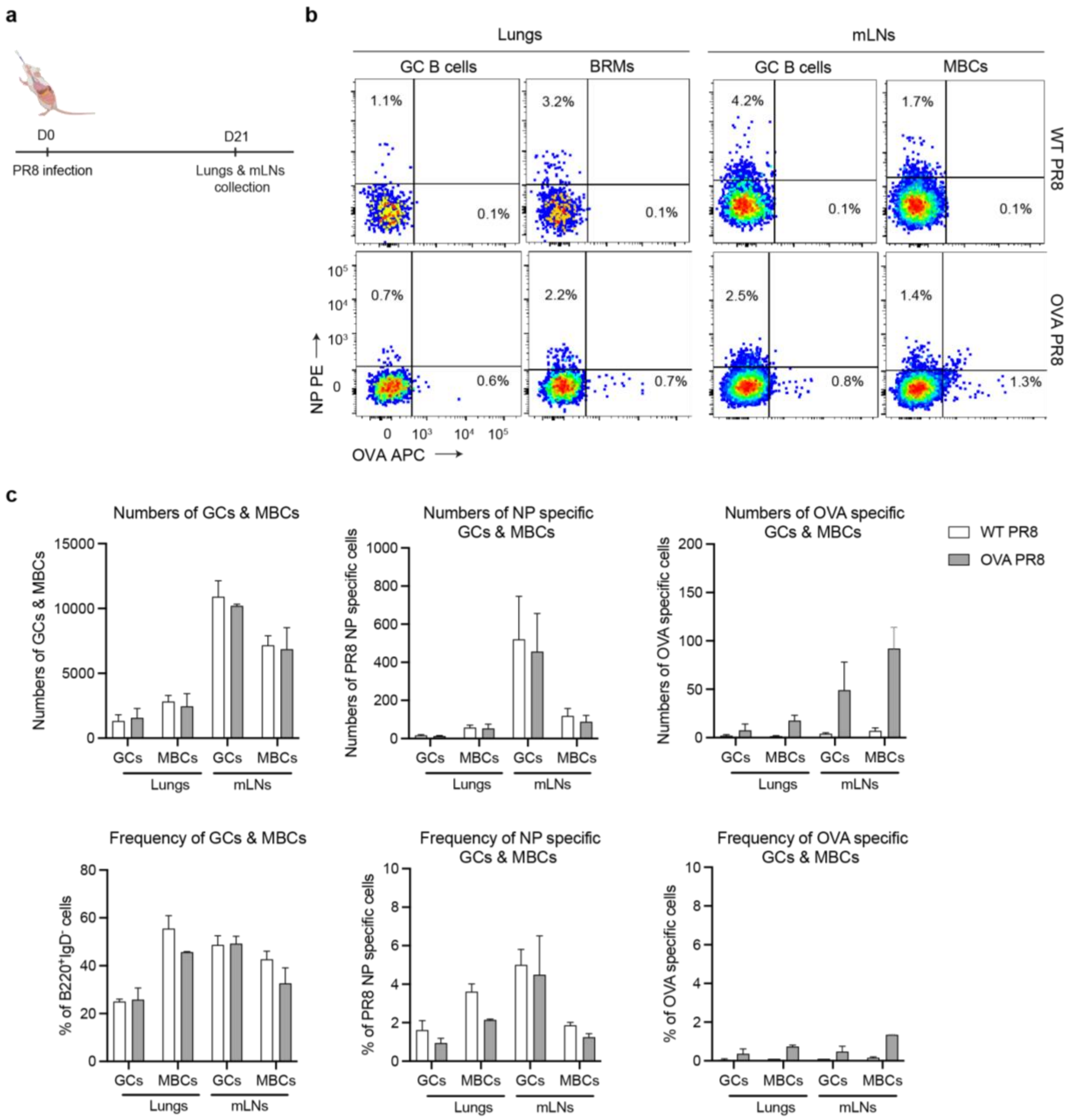

Figure S8: Establishment of antigen-specific B cell population in lungs and mLNs following OVA PR8 infection. **a**). Groups of C57BL/6 were i.n. infected with WT OVA PR8 at $10^{5.5}$ pfu. At day 21 post infection, lungs and mLNs were harvested to assess B cell responses. **b**). Representative flow plot flu- (NP-) or OVA-specific GC B cells and memory B cells in lungs and mLNs. **c**). Numbers and frequencies of total, NP- and OVA-specific GC B cells and memory B cells in lungs and mLNs.

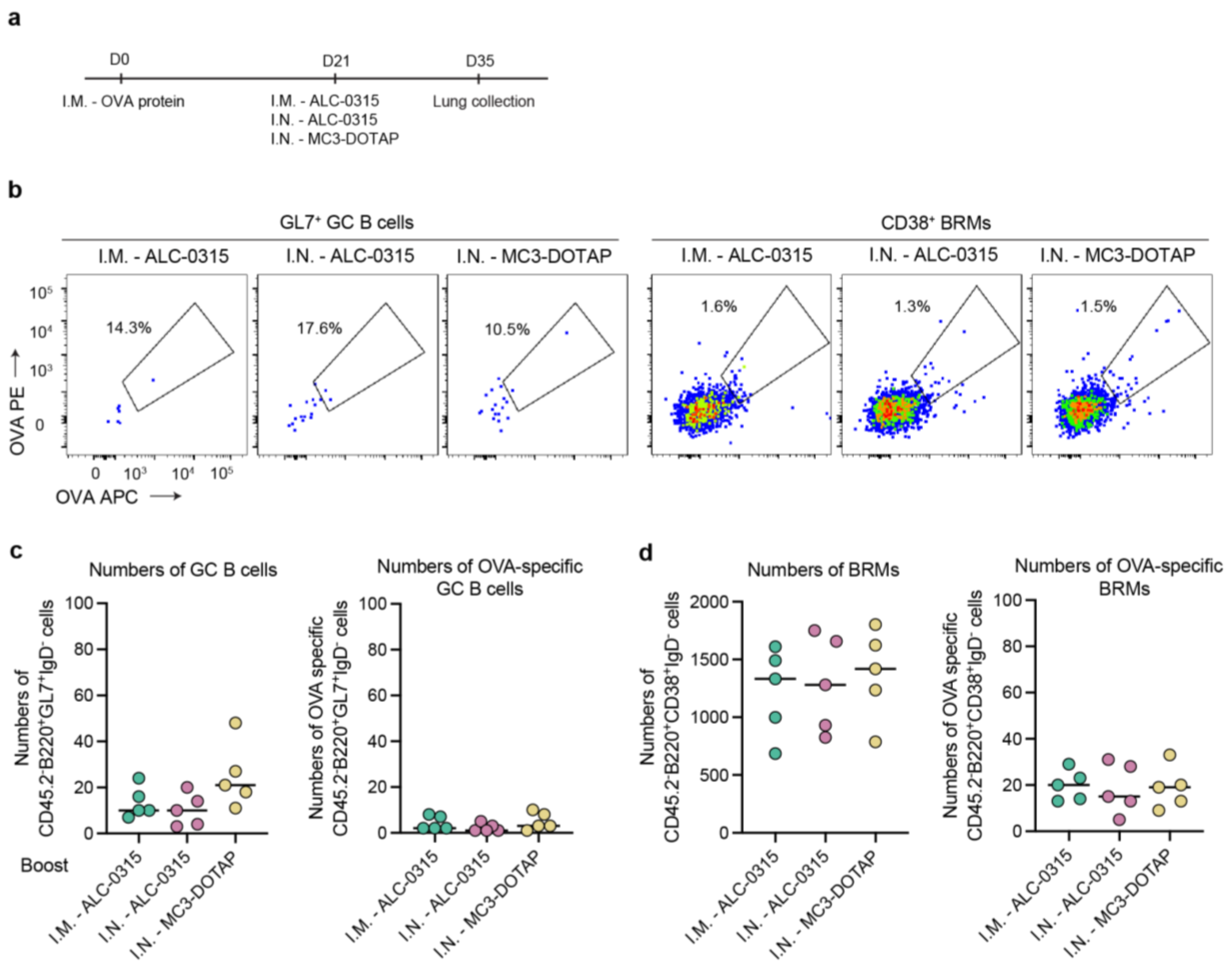

Figure S9: Minimal establishment of tissue-resident B cell responses in lungs following mRNA LNP intranasal vaccination in animals with pre-existing systemic immunity. **a**). Groups of C57BL/6 (n = 5) were i.m. primed with 5 μg OVA protein plus 50% volume of Addavax and 21 days later, i.n. boosted with OVA mRNA LNPs at 2.0 μg mRNA or i.m. boosted with ALC-0315 at 5 μg mRNA as a control. **b**) Representative flow blots of OVA-specific GC B cells and BRMs in lungs at 14 days post boost. **c**). Numbers of total and OVA-specific CD45.2-B220+IgD−GL7+ GC B cells (left) and numbers of total and OVA-specific CD45.2-B220+IgD−CD38+ BRMs (right).

Table S1: Delivery efficiency and immunogenicity of mRNA-LNPs via intramuscular and intranasal administration

**Route**		**I.M.**		**I.N.**	
Formulation		Delivery	Immunogenicity	Delivery	Immunogenicity
- DOTAP	ALC-0315	+++	+++	+++	+
	SM-102	+++	+++	+++	+
	MC3	+	+	+	+
+ DOTAP	SM-102	++	++	++	++
	MC3	+	+	++	++
